# Mechanistic insights into host adaptation, virulence and epidemiology of the phytopathogen *Xanthomonas*

**DOI:** 10.1093/femsre/fuz024

**Published:** 2019-10-03

**Authors:** Shi-Qi An, Neha Potnis, Max Dow, Frank-Jörg Vorhölter, Yong-Qiang He, Anke Becker, Doron Teper, Yi Li, Nian Wang, Leonidas Bleris, Ji-Liang Tang

**Affiliations:** National Biofilms Innovation Centre (NBIC), Biological Sciences, University of Southampton, University Road, Southampton SO17 1BJ, UK; Department of Entomology and Plant Pathology, Rouse Life Science Building, Auburn University, Auburn AL36849, USA; School of Microbiology, Food Science & Technology Building, University College Cork, Cork T12 K8AF, Ireland; MVZ Dr. Eberhard & Partner Dortmund, Brauhausstraße 4, Dortmund 44137, Germany; State Key Laboratory for Conservation and Utilization of Subtropical Agro-bioresources, College of Life Science and Technology, Guangxi University, 100 Daxue Road, Nanning 530004, Guangxi, China; Loewe Center for Synthetic Microbiology and Department of Biology, Philipps-Universität Marburg, Hans-Meerwein-Straße 6, Marburg 35032, Germany; Citrus Research and Education Center, Department of Microbiology and Cell Science, Institute of Food and Agricultural Sciences, University of Florida, 700 Experiment Station Road, Lake Alfred 33850, USA; Bioengineering Department, University of Texas at Dallas, 2851 Rutford Ave, Richardson, TX 75080, USA; Center for Systems Biology, University of Texas at Dallas, 800 W Campbell Road, Richardson, TX 75080, USA; Citrus Research and Education Center, Department of Microbiology and Cell Science, Institute of Food and Agricultural Sciences, University of Florida, 700 Experiment Station Road, Lake Alfred 33850, USA; Bioengineering Department, University of Texas at Dallas, 2851 Rutford Ave, Richardson, TX 75080, USA; Center for Systems Biology, University of Texas at Dallas, 800 W Campbell Road, Richardson, TX 75080, USA; Department of Biological Sciences, University of Texas at Dallas, 800 W Campbell Road, Richardson, TX75080, USA; State Key Laboratory for Conservation and Utilization of Subtropical Agro-bioresources, College of Life Science and Technology, Guangxi University, 100 Daxue Road, Nanning 530004, Guangxi, China

**Keywords:** plant disease, adaptation, extracellular polysaccharides, biofilm, type III effectors, regulatory circuits

## Abstract

*Xanthomonas* is a well-studied genus of bacterial plant pathogens whose members cause a variety of diseases in economically important crops worldwide. Genomic and functional studies of these phytopathogens have provided significant understanding of microbial-host interactions, bacterial virulence and host adaptation mechanisms including microbial ecology and epidemiology. In addition, several strains of *Xanthomonas* are important as producers of the extracellular polysaccharide, xanthan, used in the food and pharmaceutical industries. This polymer has also been implicated in several phases of the bacterial disease cycle. In this review, we summarise the current knowledge on the infection strategies and regulatory networks controlling virulence and adaptation mechanisms from *Xanthomonas* species and discuss the novel opportunities that this body of work has provided for disease control and plant health.

## Teaser

Here, we discuss the current knowledge surrounding regulatory networks and systems that control virulence and adaption mechanisms in *Xanthomonas* species. Additionally, we detail how study of these pathogens has provided novel opportunities for disease control and plant health.

## INTRODUCTION

**Figure 1.**
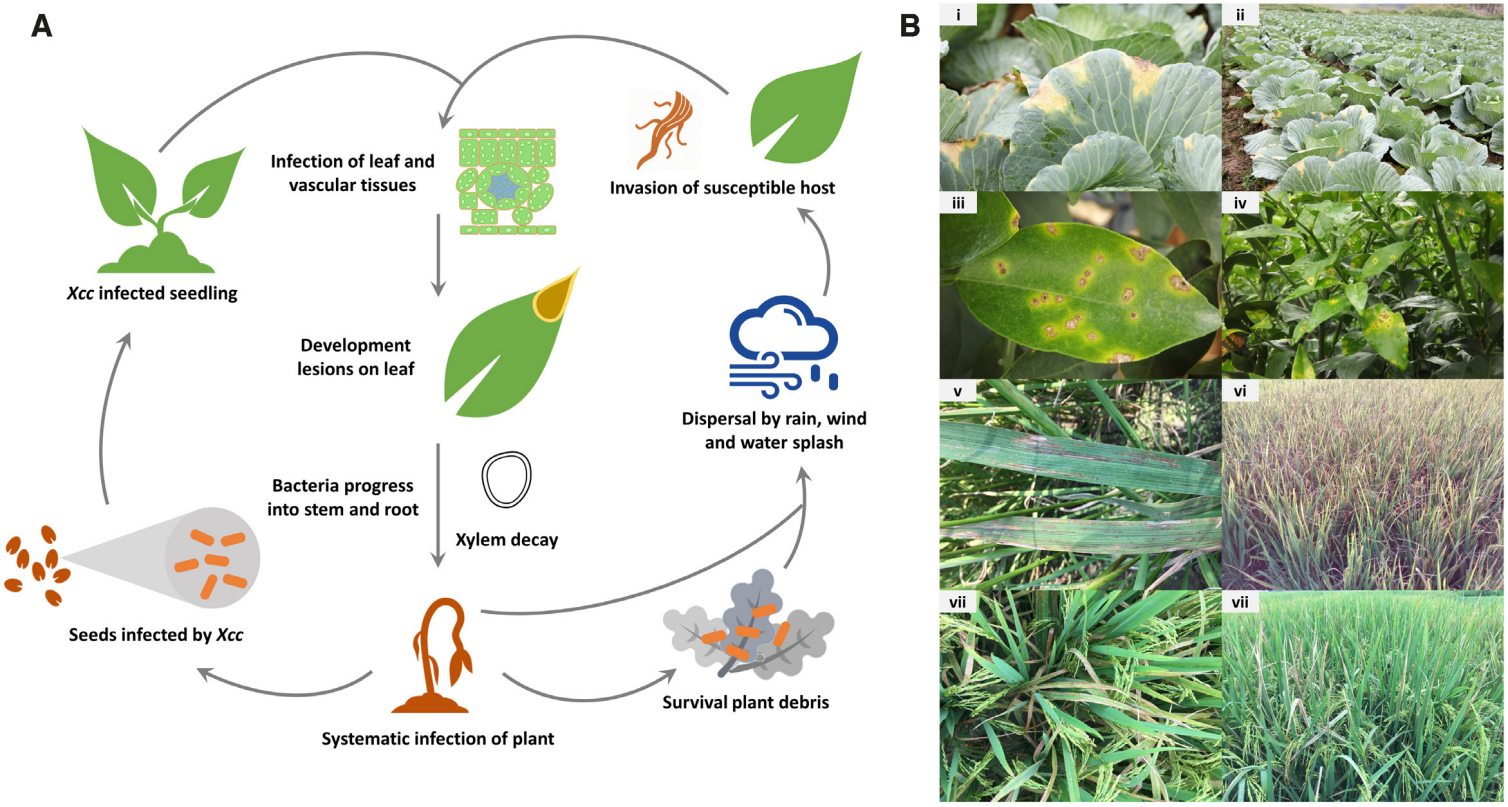
Life cycle and disease symptoms of *Xanthomonas*. **(A)**, Model illustrating the life cycle of the black rot pathogen *Xanthomonas campestris* pv. *campestris* (*Xcc*). Like most Xanthomonads, *Xcc* can survive in plant debris in soil for up to two years, but not more than six weeks in free soil. *Xcc* also has the ability to colonise plant seeds which represents a major route of disease transmission. *Xcc* can also be spread from infected plants to healthy plants by various environmental and mechanical means. After germination of colonised seeds, the seedling becomes infected. This may manifest as shrivelling and the blackening of the margins of the seedling. *Xcc* may also invade mature plants via the hydathodes, although leaf damage caused by insects and the root system also serve as portals of entry. These entry points usually provide a direct path to the plant vascular system leading to systemic host infection. V-shaped necrotic lesions extending from the leaf margins manifest as the infection develops. The disease draws its name from the blackened veins within the necrotic lesions. **(B)**, Examples of disease symptoms caused by various *Xanthomonas* species. (i, ii) Black rot of cabbage caused by *Xanthomonas campestris* pv. *campestris*. (iii, iv) Citrus canker of citrus caused by *Xanthomonas citri* pv. *citri*. (v, vi) Bacterial leaf streak of rice caused by *Xanthomonas oryzae* pv. *oryzicola*. (vii, viii) Bacterial blight of rice caused by *Xanthomonas oryzae* pv. *oryzae*.

*Xanthomonas* (two Greek words; *xanthos*, meaning ‘yellow’, and *monas*, meaning ‘entity’) is a large genus of plant-associated Gram-negative bacteria. These yellow-pigmented bacteria are generally rod shaped with a single polar flagellum, are obligate aerobes and have an optimal growth temperature of between 25 and 30°C. (Bradbury 1984) The genus, which resides at the base of the gamma subdivision of the proteobacteria (Jun *et al*. 2010), comprises 27 species that cause serious diseases in almost 400 plants (124 monocots and 268 dicots) including a wide variety of important crops such as rice, citrus, cabbage and pepper (Ryan *et al*. 2011). Pathogenic species of *Xanthomonas* show a high degree of host plant specificity and species can be further differentiated into pathovars that are defined by characteristic host range and/or tissue specificity, invading either the xylem elements of the vascular system or the intercellular spaces of the mesophyll parenchyma tissue of the host (Ryan *et al*. 2011; Jacques *et al*. 2016) (Fig. 1, Table 1). Along with host range being defined at the plant species or genus level, several *Xanthomonas* intrapathovar groups of strains interact with intraspecies variants of hosts. Races have been described within several pathovars, including *Xanthomonascampestris* pv. *campestris* and *Xanthomonas oryzae* pv*. oryzae* to group strains that interact specifically with some host cultivars near isogenic lines carrying specific resistance genes or varieties (Vicente and Holub 2013; Jacques *et al*. 2016).

**Table 1.** List of names and acronyms of*Xanthomonas* strains discussed in this review.

*Xanthomonas spp*.	Pathovar	Acronym	Host	Disease	Taxonomy ID
*X*. *albilineans*			Sugarcane	Leaf scald	NCBI:txid29447
*X*. *alfalfae*			Alfalfa	Bacterial leaf spot	NCBI:txid366650
*X*. *arboricola*	*X*. *arboricola* pv. *pruni*	*Xap*	Prunus	Bacterial spot	NCBI:txid69929
	*X. arboricola* pv*. punicae*	*Xcp*	Pomegranate (*Punica granatum*)	Leaf blight	NCBI:txid487838
	*X. arboricola* pv*. juglandis*	*Xaj*	Persian (English) walnut (*Juglans regia*)	Walnut blight	NCBI:txid195709
*X*. *axonopodis*	*X*. *axonopodis* pv. *manihotis*	*Xam*	Cassava	Bacterial blight	NCBI:txid43353
*X*. *campestris*	*X. campestris* pv. *armoraciae*	*Xca*	Horseradish	Bacterial leaf spot	NCBI:txid329463
	*X*. *campestris* pv. *campestris*	*Xcc*	Brassicaceae	Black rot	NCBI:txid340
	*X*. *campestris* pv. *leersiae*	*Xcl*	Perennial grass	Bacterial streak	NCBI:txid487875
	*X*. *campestris* pv. *musacearum*	*Xvm*	Banana	Enset wilt	NCBI:txid454958
	*X. campestris* pv*. raphani*	*Xcr*	Brassica oleracea	Bacterial leaf spot	NCBI:txid359385
	*X*. *campestris* pv. *vitians*	*Xcv*	Lettuce	Bacterial leaf spot	NCBI:txid83224
*X*. *cannabis*			Cannabis (*Cannabis sativa* L.)	Bacterial leaf spot	NCBI:txid1885674
*X. citri*	*X*. *citri* pv. *citri*	*Xcci*	Citrus	Citrus canker	NCBI:txid611301
	*X. citri* pv. *fuscans*	*Xcf*	Bean (*Phaseolus vulgaris* L.)	Bacterial blight	NCBI:txid366649
	*X. citri* pv. *glycines*	*Xcg*	Soybean (*Glycine max*)	Bacterial pustule	NCBI:txid473421
	*X. citri* pv. *malvacearum*	*Xcm*	Cotton (*Gossypium spec*.)	Bacterial blight	NCBI:txid86040
	*X. citri* pv. *mangiferaeindicae*	*Xmi*	Mango (*Mangifera indica*)	Bacterial black spot	NCBI:txid454594
	*X*. *citri* pv. *punicae*	*Xcp*	Pomegranate (*Punica granatum*)	Leaf blight	NCBI:txid487838
*X.cucurbitae*			Cucurbits	Bacterial spot	NCBI:txid56453
*X*. *cynarae*			Artichoke (*Cynara scolymus* L.)	Bacterial bract spot	NCBI:txid10214
*X*. *euvesicatoria*	*X. campetris* pv. *vesicatoria*	*Xav*	Pepper and tomato	Bacterial leaf spot	NCBI:txid456327
*X*. *floridensis*			Watercress	–	NCBI:txid1843580
*X*. *fragariae*			Strawberry	Bacterial angular leaf spot	NCBI:txid48664
*X*. *gardneri*			Pepper and tomato	Bacterial spot	NCBI:txid90270
*X*. *maliensis*			Rice	–	NCBI:txid1321368
*X*. *nasturtii*			Watercress	–	NCBI:txid1843581
*X*. *oryzae*	*X*. *oryzae* pv. *oryzae*	*Xoo*	Rice	Bacterial blight	NCBI:txid64187
	*X*. *oryzae* pv. *oryzicola*	*Xoc*	Rice	Bacterial streak	NCBI:txid129394
*X. perforans*			Tomato	Bacterial spot	NCBI:txid442694
*X*. *phaseoli*	*X*. *phaseoli* pv. *phaseoli*	*Xcp*	Bean (*Phaseolus vulgaris* L)	Bacterial blight	NCBI:txid1985254
*X. prunicola*			nectarine (Prunus persica var. nectarina) trees	–	NCBI:txid2053930
*X*. *pseudoalbilineans*	*Not in NCBI list yet*				
*X. sacchari*			Sugarcane	Chlorotic streak disease	NCBI:txid56458
*X. translucens*	*X. translucens* pv. *translucens*	*Xtt*	Wheat	Black chaff	NCBI:txid134875
	*X*. *translucens* pv. *undulosa*	*Xtu*	Wheat	Black chaff	NCBI:txid487909
*X*. *vasicola*	*X. vasicola* pv. *vasculorum*	*Xvv*	Sugarcane	Gumming disease	NCBI:txid325776

Note: *Xanthomonasarboricola* pv*. punicae* is currently listed as *X*. *citri* pv. *punicae* in NCBI taxonomy.

The main route of *Xanthomonas* species (spp.) transmission is via contaminated seeds, although weeds and infected plant debris are also potential sources of inoculum (Gitaitis and Walcott 2007). Initially, bacteria grow epiphytically (on leaf surfaces), and then enter into the host through either hydathodes or wounds to spread systemically through the vascular system or through stomata to colonise the mesophyll parenchyma (Ryan *et al*. 2011; Fig. 1). For example, *X*. *campestris* pv. *campestris* (*Xcc*) causes a systemic vascular disease of brassicas known as black rot, characterised by V-shaped chlorotic lesions spreading from the leaf margins, whereas *X. campestris* pv. *armoraciae* (*Xca*) causes a leaf spotting disease of the brassica mesophyllic tissue but does not colonise the vascular system (Fargier, Fischer-Le Saux and Manceau 2011).

Here, we review the lifestyle and properties of *Xanthomonas* spp. with an emphasis on adaptability, virulence and epidemiology. We describe the mechanisms that contribute to the ability of *Xanthomonas* spp. to survive during epiphytic and endophytic growth and to cause disease, considering the role of diverse regulatory and sensing systems, secreted effectors and the biosynthesis of extracellular polysaccharide (EPS) and lipopolysaccharides (LPS). We discuss the insight that genome sequencing of *Xanthomonas* spp., has had on taxonomical classifications of the bacteria and understanding pathogen evolution and host adaptation. Finally, we consider how recent work understanding *Xanthomonas* spp. pathophysiology is being exploited for disease suppression and biocontrol.

## XANTHOMONAS LIFESTYLE

### *Xanthomonas* in the plant microbiome

*Xanthomonas* is known to live part of its life cycle outside of the plant host such as in the lesion of fallen leaves or freely in the soil, which might serve as an inoculum for further infection of plant hosts (Zhao, Damicone and Bender 2002). Multiple studies, covering numerous hosts and geographic locations, have identified the *Xanthomonadales* order as an important component in both the rhizosphere and soil microbiome environments, where they compose between 2% and 7% of the bacteria in the microbial community (Bulgarelli *et al*. 2015; Bhattacharyya *et al*. 2018). However, metagenomic analysis at higher resolution indicated that the abundance of the *Xanthomonas* genus ranges from below detection levels to 0.7% in the rhizosphere and soil microbiomes (Souza *et al*. 2016; Xu *et al*. 2018).

The abundance of *Xanthomonas* within the plant phyllosphere microbial community varies greatly between plant species, tissues, sampling season and geographic locations. *Xanthomonas* has been identified in the aerial parts of tomato, lettuce, rapeseed, clover, soybean, arabidopsis and rice (Delmotte *et al*. 2009; Knief *et al*. 2012; Rastogi *et al*. 2012; Ottesen *et al*. 2019). Several metagenomics analyses from the United States recognise *Xanthomonas* as a component of the tomato phyllosphere (Ottesen *et al*. 2015, 2019). Anatomical based analysis in tomato plants also identified *Xanthomonas* as an important component of the tomato microbiome (Ottesen *et al*. 2013). Notably, *Xanthomonas* represented 10%–40% of the whole bacterial communities of fruits, leaves and flowers, while, similarly to other studies, was not found in a significant abundance in the rhizosphere (Ottesen *et al*. 2013). *Xanthomonas* was also found as a dominant member in the microbiome of field grown Romain lettuce phyllosphere (Rastogi *et al*. 2012; Burch *et al*. 2016). In particular, one large scale microbiome study with samples collected from 88 different lettuce growing areas has identified *Xanthomonas* to be present in about a third of all samples, where it comprises, on average, 4% of the lettuce microbiome (Rastogi *et al*. 2012). It should be noted that in many cases *Xanthomonas* was not found as a major component of the phyllosphere, indicating that *Xanthomonas* is not an integral component of the core plant microbial community (Liu *et al*. 2017; Suhaimi *et al*. 2017).

### Epiphytic lifestyle

The infection cycle of *Xanthomonas* can be divided into the epiphytic stage and the endophytic stage. The epiphytic stage initiates once bacteria are introduced into the aerial tissues of a new host, usually leaf or fruit tissue and continues until the entrance into the host tissue via the plant natural openings and wounds. Once inside a host plant, the bacteria enter the endophytic stage and colonise the host. Upon reaching high population bacteria re-emerge onto the leaf surface and are transmitted mostly through wind or rain to a new host starting the infection cycle again (Moreira *et al*. 2015). Many aspects of the *Xanthomonas* endophytic infection have been studied and factors that are important have been described (see below). In contrast, less attention has been given to the investigation of the epiphytic stage of the *Xanthomonas* lifestyle.

Traditionally, the plant surface area is considered to be a hostile environment for bacteria (Lindow and Brandl 2003). In this environment bacteria are exposed to severe radiation, unstable humidity, limited nutrient resources and competition with other bacteria occupying the same niche. In spite of these obstacles, *Xanthomonas* can sustain in the leaf surface areas for extended periods of time (Rigano *et al*. 2007b; Zarei *et al*. 2018). *Xanthomonas* strains were reported to be capable of maintaining stable bacterial populations in their host or non-host plant. Studies comparing epiphytic survival in host and non-host plant in different pathosystems reported better epiphytic fitness in the host, indicating that host specificity plays a role in epiphytic fitness (Rigano *et al*. 2007b; Zarei *et al*. 2018). The duration that *Xanthomonas* bacteria can sustain on the leaf surface is less clear and the survival ranges between a few weeks to several months in different reports (Rigano *et al*. 2007b; Zarei *et al*. 2018). We speculate that these variations may result from the use of different bacterial species, host plants or field environments.

### Attachment and biofilm formation on the plant surface

When introduced to the plant surface *Xanthomonas* utilises multiple adhesion strategies to attach to the plant surface, including bacterial surface polysaccharides (Vorhölter, Niehaus and Pühler 2001; Petrocelli *et al*. 2012), adhesion proteins (Pradhan, Ranjan and Chatterjee 2012) and the type IV pilus (Dunger *et al*. 2014; Petrocelli *et al*. 2016). After initial attachment, *Xanthomonas* forms biofilm-like structures. Similar structures were found in a multitude of leaf surface dwelling bacteria and it was hypothesized that creation of a microenvironment within the leaf surface biofilm protects the bacteria from harsh abiotic stress conditions in the phyllosphere (Yu *et al*. 2013). Biofilm is a general term for structures created by bacterial communities on the surface areas and composed of bacterial cells connected through extracellular polymeric substance matrix composed of exopolysaccharides (EPS), extracellular DNA (eDNA), proteins and lipids (Castiblanco and Sundin 2016). *Xanthomonas* biofilm is a dynamic structure and its assembly and dispersal is mediated by the quorum sensing signal molecule diffusible signal factor (DSF). Through the internal second messenger cyclic di-GMP, DSF promotes biofilm formation through induction of EPS production and pilus assembly (Tang *et al*. 1991; Guo *et al*. 2011; Rai *et al*. 2012; Ryan 2013). In parallel, DSF negatively controls the biofilm by positively regulating β-1,4-mannanase, ManA, which disperses EPS and disassembles biofilm (Dow *et al*. 2003; Tao, Swarup and Zhang 2010; Chen *et al*. 2010b).

The biofilm matrix in *Xanthomonas* spp. is considered to be mainly composed of Xanthan gum (discussed in detail below) (Crossman and Dow 2004). Xanthan gum biosynthesis is a complex procedure mediated by the *gum* operon, a large transcriptional unit containing 12 enzyme coding genes (*gumB-gumM*) (Vojnov *et al*. 2001a). Disruption of either *gumB*, *gumC*, *gumD*, *gumE*, *gumH*, *gumK*, *gumI* and *gumK* compromises both Xanthan gum production (Vojnov *et al*. 1998; Kim *et al*. 2009) and biofilm formation (Rigano *et al*. 2007b; Li and Wang 2011a). In addition to the *gum* cluster, biofilm EPS composition was identified to be dependent on *xagABC*, a glycosyltransferase system associated with polysaccharide biosynthesis (Tao, Swarup and Zhang 2010; Li and Wang 2012). eDNA has long been considered to be an integral part in bacterial biofilms (Okshevsky and Meyer 2015) but only recently shown to play a role in *Xanthomonas* biofilm formation. Specifically, Sena-Vélez and colleagues have shown that eDNA is highly abundant in *Xanthomonas citri* mature biofilm and accumulates throughout biofilm development (Sena-Vélez *et al*. 2016). In addition, extracellular DNase treatment was able to inhibit biofilm formation but only partially dispersed mature biofilm, suggesting that eDNA plays a significant role in early stage of biofilm establishment (Sena-Vélez *et al*. 2016). Other factors that were reported to be critical for *Xanthomonas* biofilm formation include cell surface LPS and lipoproteins (Li and Wang 2011a; Li and Wang 2011b; Petrocelli *et al*. 2012; Yan, Hu and Wang 2012), the bacterial flagellum and type IV pili (Malamud *et al*. 2011; Dunger *et al*. 2014).

While biofilm formation, stability and composition have been extensively examined in *Xanthomonas* spp., very few studies directly tested the effect of biofilm disruption on epiphytic fitness or analysed biofilm composition under environmental conditions. EPS biosynthesis deficient mutants (*gumB* and *gumD*, in particular) displayed significant reduction in leaf surface survival compared to the wild type *X. citri* pv*. citri* (*Xcci)* and *X. axonopodis* pv*. manihotis* (*Xam*) (Kemp *et al*. 2004; Dunger *et al*. 2007; Rigano *et al*. 2007b). Furthermore, Li and Wang have demonstrated that chemical inhibition of biofilm formation by D-leucine and 3-indolylacetonitrile disrupts leaf surface colonisation and disease development by *Xcci* (Li and Wang 2013).

### Surface movement and chemotaxis

Only a handful of studies have focused on motility and chemotactic movement on the plant aerial surfaces by bacterial pathogens (Hockett, Burch and Lindow 2013; Moreira *et al*. 2015) and the importance of chemotactic motility in the epiphytic environment is not completely clear. It is hypothesized that motility is required for clustering of bacteria on the leaf surface and directing the bacteria towards potential entry points into the plant (Hockett, Burch and Lindow 2013; Moreira *et al*. 2015).

A few studies have monitored GFP-labelled *Xanthomonas* on host leaves, showing similar patterns of behaviour. A few hours after inoculation, attachment of single bacterial colonies can be observed on the leaf surface. In the following days, bacteria start clustering together in microcolonies and later aggregate in the proximity of plant natural openings such as stomata or hydathodes, then enter the host endophytic spaces (Zhang *et al*. 2009; Cubero *et al*. 2011; Cerutti *et al*. 2017). Different *Xanthomonas* spp. have preferences for particular plant openings according to their colonisation strategy (Ryan *et al*. 2011). Non-systemic apoplastic *Xanthomonas* such as *X. euvesicatoria* or *X. citri* display high preference for stomata while systemic xylem colonising species like *Xcc* or *X. oryzae* pv. *oryzae* (*Xoo*) usually cluster around the hydathodes (Shekhawat and Srivastava 1972; Zhang *et al*. 2009; Cubero *et al*. 2011; Cerutti *et al*. 2017).

Directional movement of the bacteria to the plant openings is potentially mediated through chemotaxis. Chemotactic movement of *X. oryzae* towards rice xylem sap, xylem specific sugars and certain amino acids was demonstrated previously (Kumar Verma, Samal and Chatterjee 2018). Kumar Verma *et al*. have linked xylem sap- and amino acid-triggered movement to the MCP2 chemoreceptor. Deletion of either *mcp2* or chemosensory signalling protein coding genes *cheW2, cheA2, cheY1* and *cheB2* disrupts chemotactic movement towards these metabolites (Kumar Verma, Samal and Chatterjee 2018). In addition these mutants were not able to epiphytically infect rice leaves (Kumar Verma, Samal and Chatterjee 2018), indicating that *X. oryzae* uses xylem chemotactic signals leaked from hydathodes to direct themselves into the xylem vessels.

Most *Xanthomonas* spp. harbour motility-associated apparatuses like flagella and type IV pili and motility of these bacteria *in vitro* was reported to be mostly attributed to flagella (Yang *et al*. 2009; Malamud *et al*. 2011; Tian *et al*. 2015). It is yet to be shown if flagella directly play a role in endophytic fitness or plant surface motility but it was reported to be significant to infectivity (Malamud *et al*. 2011; Bae *et al*. 2018; Kumar Verma, Samal and Chatterjee 2018), suggesting that flagellar motility might play a role in the epiphytic phase.

### Response to radiation and light

On the leaf surface, bacteria are exposed to solar radiation, which includes UV-A and UV-B wavelengths that cause damage to DNA, membranes and proteins. Epiphytic bacteria adopted multiple strategies to reduce radiation exposure and its damage by blocking radiation through pigments and biofilm structures, and acquiring DNA repair complexes (Jacobs, Carroll and Sundin 2005; Cazorla *et al*. 2008). Most *Xanthomonas* spp. produce yellow membrane bound pigments called xanthomonadins, whose synthesis is dependent on a gene cluster encoding seven transcriptional units (*pigA-pigG*) (Poplawsky, Kawalek and Schaad 1993). Deletion of *pigC* and *pigG* in *X. campestris* resulted in enhanced susceptibility to radiation from natural light but not to UV (Poplawsky *et al*. 2000). Several UV sensitive mutants were identified in different *Xanthomonas* strains; most are related to response to oxidative stress and DNA repair (Martínez *et al*. 1997; Shen *et al*. 2007; Tondo *et al*. 2016).

It was reported that response to visible light plays a significant role in modulating pathogenicity and epiphytic fitness. XccBphP of *X. campestris* encodes a functional bacteriophytochrome protein displaying altered response between red and far red (Otero *et al*. 2016). Characterisation of WT and *XccBphP* mutant in light and dark conditions revealed that light negatively regulates epiphytic survival associated traits such as motility, xanthan production and extracellular hydrolase activity in a *XccBphP* dependent manner (Bonomi *et al*. 2016). LOV (Light, Oxygen, Voltage), a blue light photosensory protein of *X. citri*, was reported to regulate surface attachment and biofilm formation in a light dependent manner (Kraiselburd *et al*. 2012). Interestingly, Kraiselburd *et al*. (2012) reported that light plays a positive role in biofilm formation in *X. citri*, suggesting that the response to light is not the same in different *Xanthomonas* spp.

### Interaction with other bacteria on the aerial surfaces

Plant aerial surfaces are colonised by a multitude of microorganisms that form a complex communal structure. The interactions between different bacteria on the leaf surface have not been extensively examined, however. Similarly to other leaf surface habitating bacteria like *Pseudomonas syringae* and *Pantoea agglomerans* (Monier and Lindow 2005), aggregates of *Xanthomonas* are usually considered as a single species aggregates (Monier and Lindow, 2005). *Xanthomonas* utilizes the type IV and type VI secretion systems to interact with the microflora of the phyllosphere. *Xanthomonas citri* utilizes a type IV secretion system to kill other Gram-negative bacteria through delivery of antibacterial proteins (Souza *et al*. 2015). The group also reported that the type IV secretion system contributes to competitive fitness when inoculated with other bacteria, indicating that *Xanthomonas* utilises this system to establish itself on the leaf surface by actively killing bacteria that occupy the same niche (Souza *et al*. 2015). The same group also recently reported that the *X. citri* type VI secretion system is required for protection against the predatory amoeba *Dictyostelium* (Bayer-Santos *et al*. 2018). These studies indicate that *Xanthomonas* relies on multiple systems to respond to microorganisms in its habitat.

### Cuticle interactions, resource utilisation and metabolic activity on the plant aerial surfaces

Microorganisms on plant aerial surfaces are exposed to limited amount of macro- and micro- nutrients and unstable humidity (Lindow and Brandl 2003). A few studies have examined *Xanthomonas* metabolism on the leaf surface. Cubero and colleagues monitored the expression of an unstable GFP variant in *X. citri* colonising leaf and fruit surfaces as a marker for metabolic activity (Cubero *et al*. 2011). The study concluded that although metabolic activity was significantly reduced during the incubation on the leaf surface, it increased once bacteria reached the stomatal area (Cubero *et al*. 2011). Epiphytic survival studies do occasionally show an increase in the bacterial population on the leaf surface (Zarei *et al*. 2018), indicating that the bacteria can utilize the scarce resources in the phyllosphere to some extent. It is unclear, however, what metabolic pathways are induced in *Xanthomonas* in this environment. Déjean and colleagues reported that disruption of xylanase-coding genes in *X. campestris* resulted in reduced epiphytic growth on the surface of host and non-host plants, suggesting that xylan is available to the bacteria and can be utilised as a carbon source on the plant aerial surfaces (Déjean *et al*. 2013).

Leaf and fruit epidermal surfaces are covered by the cuticle, a waxy layer that prohibits water evaporation and serves as a physical barrier against microbial pathogens, preventing microorganisms from both entering the plant and accessing the plant water and nutrient resources (Ziv *et al*. 2018). Alteration and disruption of cuticle structure was reported in several microorganisms. *Pseudomonas syringae* bacteria secrete multiple surfactants and toxins to increase leaf wettability while multiple fungal pathogens actively degrade host cutin polymer using cutinases, lipolytic/esterolytic enzymes (Lindow and Brandl 2003). No data are available regarding *Xanthomonas*-cuticle interactions and further research is required to establish the strategies utilised by *Xanthomonas* to alter or disrupt the plant cuticle.

## XANTHOMONAS DISPERSAL AND SPREAD

### Environmental factors

Dispersal of pathogens and the transfer between hosts play an extremely important role in the pathogen infection cycle. Environmental factors that facilitate pathogen dispersal can dictate why susceptible crops are routinely infected in some areas but stay pathogen free in others. *Xanthomonas* spp. thrive better on slightly higher temperatures (on average 25–35°C) and are capable to reach high enough inoculum in the host to allow dispersal (Christiano *et al*. 2009; Morales *et al*. 2018a; 2018b). In addition, high humidity was found to be required for epiphytic survival and transmission (Christiano *et al*. 2009). In this condition, the high abundance of bacteria on the surface area of a infected host will be a more significant inoculum source for dispersal, while, in parallel, the same condition will contribute to epiphytic survival of the bacteria upon arrival to a leaf of a new host.

Plant response to warm and humid environment exacerbate disease necrotic symptoms, which increases the availability of the pathogen to the outside environment (Diab *et al*. 1982; Kumar *et al*. 2006). In high humidity, plants open stomata and hydathodes to sustain water homoeostasis (Zeng, Melotto and He 2010; Singh 2013). This open state allows both *Xanthomonas* entry from the plant surface (Zeng, Melotto and He 2010) and exit of high inoculum of bacteria from infected plants. Guttation of xylem sap from hydathodes at night and during high humidity is considered as the route of exit and entry of xylem-inhabiting *Xanthomonas* and plays a significant role in acquisition and dispersal of these pathogens (Guo and Leach 1989; Cerutti *et al*. 2017).

### Dispersal through wind and rain

Wind and rain are considered as the main carriers of most *Xanthomonas*s spp. in short- and long-distance dispersal of most *Xanthomonas* spp. Tropical storms, tornadoes and heavy rainfalls are considered as significant facilitators of long distance dispersal of many *Xanthomonas* spp. and were found to be highly correlated to outbreaks (Gottwald and Irey 2007; Champoiseau *et al*. 2009; Jha and Sonti 2009). The effect of wind on pathogen dispersal has been extensively studied in regard to citrus canker, caused by *X. citri*. Multiple epidemiological studies have identified that physical proximity to infection areas and rainfall significantly increase disease occurrence (Gottwald *et al*. 2002; Gottwald *et al*. 2007). Several studies have focused on pathogen dispersal during simulated wind and rain in laboratory conditions. These studies demonstrated a direct correlation between wind intensity and the pathogen dispersal distance between infected and neighbouring plants in *X. citri* and *X. alfalfae*, indicating that wind is a major abiotic vector of the bacteria (Bock *et al*. 2012). The understanding of the importance of wind and rain to dispersal of *Xanthomonas* has greatly contributed to disease management of citrus canker. Application of windbreaks successfully reduced pathogen spread between groves (Behlau *et al*. 2008; Moschini *et al*. 2014). In addition, tighter surveillance for canker symptomatic plants after heavy rain and storms has enabled early detection of potential outbreaks (Gottwald and Irey 2007; Canteros, Gochez and Moschini 2017).

### Dispersal by people and non-specific vectors

Agricultural practices are a major means for pathogen transmission. While it has been claimed that people and agricultural machinery are one of the main sources of short distance and long-distance transmission, few studies have been dedicated to establishing the exact effect of these factors on *Xanthomonas* dispersal. Dispersal through people and farming equipment was reported as the major source of spread of the banana and plantain pathogen *X.campestris* pv. *musacearum* (*Xvm*) (Nakato, Mahuku and Coutinho 2018). The bacteria were reported to survive on farming equipment for between one and three weeks and shown to be actively transferred from one plant to another through farming tools (Addis *et al*. 2010; Nakato, Mahuku and Coutinho 2018). Education of local farmers about the importance of cleaning of farming tools and proper destruction of infected crops is currently considered a key element in the prevention of banana *Xanthomonas* wilt in east and central Africa (Nakato, Mahuku and Coutinho 2018).

The lifecycle of several vascular tissue-residing plant pathogens such as *Candidatus* Liberibacter, *Xylella fastidiosa* and *Pantoea stewartii* includes a phase of habitation inside or associated with a specific insect vector which allows plant-to-plant transmission (Chatterjee, Almeida and Lindow 2008; Roper 2011; Wang *et al*. 2017). A similar mechanism is not known for any *Xanthomonas* spp. although speculation of a role of non-specific biotic vector has arisen (Belasque *et al*. 2005; Zandjanakou-Tachin *et al*. 2007). *Xanthomonas* bacteria have been isolated from field specimens of potential biotic vectors such as insects, birds and bats; the bacteria were shown to survive on these alleged vectors for a duration of a few days (Belasque *et al*. 2005; Zandjanakou-Tachin *et al*. 2007). Studies of active plant-to-plant transmission conducted with leafminers, flea beetles and grasshoppers have shown that insect to plant transmission of *Xanthomonas* spp. can occur but in a very low capacity, indicating that vector transmission probably only plays a relatively small role in short distance transmission (Belasque *et al*. 2005; Zandjanakou-Tachin *et al*. 2007; van der Wolf and van der Zouwen 2010; Buregyeya *et al*. 2014). Van der Wolf and colleagues showed that *X. campestris* can be transmitted to cabbage flowers though flies and bumble bees and demonstrated that flower infection can be carried on to the seeds (van der Wolf and van der Zouwen 2010; van der Wolf *et al*. 2019). The prevalence of such transmission in the field is still unknown and should be subjected to further study. Transmission through pollinating vectors is speculated to be one of the main means of dispersal for *Xvm* (Nakato, Mahuku and Coutinho 2018). It was found that stingless bees and fruit flies carry high bacterial inoculum of *Xvm* when isolated from infected fields (Tinzaara *et al*. 2006) and that floral parts are usually the initial inoculation point of the bacteria (Ocimati *et al*. 2012; Rutikanga *et al*. 2015). Following these observations, floral debudding is now commonly practiced in banana to prevent pathogen infection through any insect vector (Blomme *et al*. 2017). It should be noted that while insect infection is very likely linked to *Xvm* dispersal, direct vector-mediated plant-to-plant transmission has yet to be experimentally verified. Future research of this unique system can provide new insights into *Xanthomonas*-vector interactions.

### Seed dispersal

Seed transmission is reported for many *Xanthomonas* spp. and contaminated seeds are considered as a dominant factor in epidemics of black rot of crucifers, bacterial spot of tomato, bacterial blight of cassava and many others (Gitaitis and Walcott 2007). Although a significant amount of work has been dedicated towards standardising screening methods for identification of infected seeds and general seed treatment (Gitaitis and Walcott 2007), our understanding of the mechanisms of seed transport and of the bacterial lifestyle within the seed is fragmentary. *Xanthomonas* spp. can survive inside seeds for periods of several weeks to several months (He and Munkvold 2013) and studies of *X. campestris* and *X. oryzae* have also detected that duration of survival is different between seeds from different hosts (Dutta *et al*. 2016). Transmission efficiency from seed to seedling for several *Xanthomonas* spp. was found to be directly dependent on the amount of bacteria inoculum in the seed (Darrasse *et al*. 2007). Transmission from plant to seeds usually occurs when plants are heavily infected but the frequency appears to be pathogen and host dependent, varying from less than 1% to around 90% (Humeau *et al*. 2006; Giovanardi *et al*. 2015). No consistent data is available regarding which part of the seed is contaminated by the bacteria. Contamination of the outer seed coat was reported in most cases while contamination of the inner seed and embryo was reported in some cases but not others. For instance, only external colonisation was reported in lettuce seeds infected with *X. campestris* pv. *vitians* (*Xcv*) (Barak, Koike and Gilbertson 2002) while *Xcc*, *X. euvesicatoria* and *X. citri* pv. *fuscans* (Xcf) were reported to colonise the inner seeds of cauliflower, tomato and bean, respectively (Sharma and Agrawal 2014; Darrasse *et al*. 2018; van der Wolf *et al*. 2019). Plant to seed transmission has long been speculated to originate from infected reproductive organs. Recent work by van der Wolf and colleagues (2013) has indeed identified a direct link between floral infection and inner seed colonisation. Cauliflower plants were spray inoculated with *X. campestris* at different developmental stages and seed colonisation was monitored. Symptoms were apparent in plants that were inoculated in all developmental stages but seed colonisation was only seen when plants were inoculated during flowering time (van der Wolf, van der Zouwen and van der Heijden 2013). In a further study using GFP-labelled *X. campestris*, the group demonstrated that the bacteria can successfully colonise and cause symptoms in siliques and subsequently colonise both the outer seed coat and the endosperm and embryo (van der Wolf *et al*. 2019). This data lead to the hypothesis that infected reproductive tissues later develop into infected endosperms and embryos. The group also estimated that reproductive tissues are externally infected through water splash or pollinators.

## PATHOGENOMICS OF XANTHOMONADS

**Figure 2.**
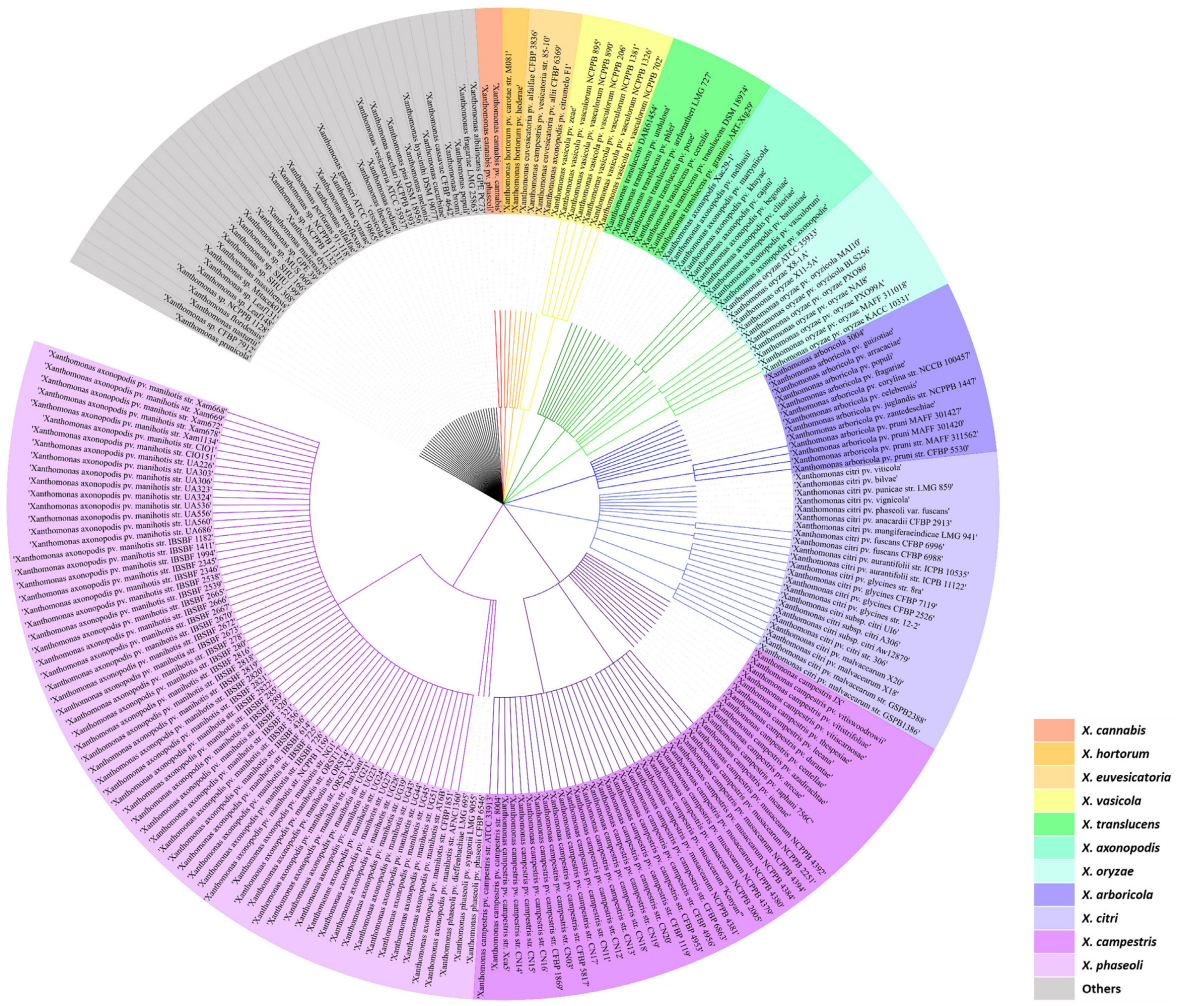
Phylogenetic tree of the Xanthomonas genus based on the NCBI taxonomy. The list of taxonomic names obtained from the NCBI Taxonomy Browser filtered using ‘has genome sequences’. The tree was annotated and visualized using iTOL (Interactive Tree Of Life) software.

There are ∼1200 genomes of *Xanthomonas* available on NCBI genome browser, which represents a 21-fold increase compared to the 2011 report (Fig.   2) (Ryan *et al*. 2011). This wealth of genome data has revolutionised our understanding of bacterial taxonomy and pathogen evolution, as well as factors contributing to virulence and host adaptation. In this section, we discuss case studies/examples that have advanced our understanding of this pathogen.

### Taxonomic re-classifications with the knowledge of sequenced genomes

Availability of whole genome sequences for the majority of *Xanthomonas* spp. has allowed the expansion of our understanding of the overall relationships among different species beyond classically used multilocus sequence typing. Average nucleotide identity among representative strains has been used as a criterion for classifying strains into species (Konstantinidis and Tiedje 2005). Several taxonomic revisions have been proposed in a larger species complex, sp. *axonopodis*, sp. *campestris*. While species nomenclature in xanthomonads has been defined based on its host specificity, whole genome comparisons have led to redefinition of some species. For example, *Xanthomonas gardneri* and *Xanthomonas cynarae* were recently placed into a single species (Timilsina *et al*. 2019). Additionally, several species of *Xanthomonas* (*Xanthomonas nasturtii* sp. nov. and *Xanthomonas floridensis* sp. nov.) associated with watercress production in Florida were defined. Sequencing genomes of diverse strains belonging to closely related species infecting tomato and/or pepper indicated the admixture of strains arising due to frequent genetic exchange among different strains of the closely related species (Barak, Koike and Gilbertson 2002; Jibrin *et al*. 2018). These hybrid strains pose a challenge to the current taxonomic definitions of a species based on average nucleotide identity (ANI). Although their ANI values are above 96%, these strains do differ in many phenotypic tests.

### Evolutionary histories of pathogenicity factors

Comparative genomics of the occurrence of the type III secretion system (T3SS) has revealed that the Hrp2 family T3SS (for Hypersensitive Resistance and Pathogenicity) is present in the majority of xanthomonads, with an exception of *X. albilineans* that in contrast possesses a SPI-1 T3SS system. Four species, *Xanthomonas sacchari, Xanthomonas cannabis, Xanthomonas pseudoalbilineans* and *Xanthomonas maliensis*, lack any T3SS and associated effectors. *Xanthomonas arboricola* strains lacking a T3SS were considered as commensals. Given the relevance of evolution of the T3SS in ecological adaptation of pathogens, Merda *et al*. (2017) studied evolutionary history of Hrp2 family T3SS and associated effectors (T3Es) in xanthomonads. T3SS loss/acquisition events as well as genomic rearrangements in T3SS-containing region were compared across 82 genome sequences representing the diversity of the *Xanthomonas* genus (Merda *et al*. 2017). This study inferred three ancestral acquisitions of this cluster during *Xanthomonas* evolution followed by subsequent losses in some commensal strains and re-acquisition in some species. Several genes present in the Hrp2 cluster were also influenced by interspecies homologous recombination indicating large fragment recombination in this cluster. Interestingly, HGT of the entire cluster was also observed. It was speculated that loss of T3SS in some of *X. arboricola* commensals could be due to a high fitness cost associated with maintenance (Merda *et al*. 2017). These commensals could also act as profiteers given their simultaneous isolation with pathogenic species possessing a T3SS. Interestingly, the species *X. arboricola* contains strains that possess T3SS and variable numbers of T3Es as well as commensal strains lacking T3SS but possessing four effectors (*xopF1*, *xopM*, *avrBs2* and *xopR*). The expansion of genome sequence data in the recent years has challenged the concept of a ‘core effectorome’ of *Xanthomonas* that comprises 10 T3Es; this ‘core’ is now thought to comprise only four effectors defined as the reduced ancestral core T3SE repertoire (Merda *et al*. 2017). These four effectors in commensal strains may allow them to overcome basal immune responses. Intriguingly, Merda *et al*. (2017) also identified a few *X. arboricola* strains lacking a T3SS but possessing two effectors, *avrBs2* and *xopR*. It remains to be investigated whether these two effectors are secreted into the host and if so, whether these effectors have additional function independent of T3SS. It has been proposed that pathogenic strains that have accumulated other ‘previously defined core’ T3Es (*xopL*, *xopN*, *xopQ*, *xopX* and *xopZ*) define a basal step of pathogen emergence since these effectors have been shown to target pattern-triggered immunity (PTI) of plants (Merda *et al*. 2017).

### Host adaptation/host and tissue specificity

Bacteria within the genus *Xanthomonas* are known for host specificity and the species were defined and classified based on their host range (Dye 1958). Studies involving identification of genetic determinants of host specificity in xanthomonads have been accelerated due to whole genome sequencing of closely related strains/lineages showing differences in phenotypes on certain hosts. Hajri *et al*. proposed ‘repertoire-for-repertoire hypothesis’ by which repertoires of T3Es in different species of *Xanthomonas* were proposed as determinants of host specificity (Table S1, Supporting Information) (Hajri *et al*. 2009). Chen *et al*. (2018) studied genes encoding transcription activator-like effectors (*tal* genes) from 17 *Xanthomonas* strains that cause common bacterial blight of beans, representing the four genetic lineages of *Xcf* and *X. phaseoli* pv. *phaseoli* (*Xcp*). Two chromosomic *tal* genes (*tal20F* and *tal18*) and two *tal* genes (*tal23A*, *tal18H*) encoded on plasmids were shared among most of phylogenetically distinct *Xcf* and *Xcp* strains. Since *tal23A* homologues genes are conserved in all *Xanthomonas* strains responsible for the common bacterial blight disease, this gene could be crucial in *Xcf* and *Xcp* pathogenicity and adaptation to common bean. These findings support the contention that horizontal transfer of plasmid-borne *tal* genes between distant lineages contributes to host adaptation (Chen *et al*. 2018).

Analysis of the complete genome sequence of the *X. albilineans* pathogenic strain GPE PC73 has allowed identification of several strategies specific to this strain to allow spread within sugarcane xylem vessels (Pieretti *et al*. 2009). Most of the known pathogenicity/virulence factors from other *Xanthomonas* strains are conserved in *X. albilineans* with the exception of the xanthan gum biosynthesis cluster and the T3SS system Hrp 2. The absence of any Hrp cluster can be a result of reductive genome evolution to live and multiply in a dead cell environment (the xylem). The presence of a T3SS belonging to the SP-1 injectisome family may in contrast be involved in the successful colonisation of the leaf surface by the pathogen. A key feature of *X. albilineans* is the ability to colonise a nutrient poor environment. Pieretti and colleagues identified 35 TonB-dependent transporters encoded by the chromosome of the *X. albilineans* strain GPE PC73 which could be important in active transport of plant-derived nutrients. In addition, 12 genes that encode for non-ribosomal peptide synthetases (NRPs) comprising 4% of the chromosome were identified. Nine of these genes are present in the genomes of *Xanthomonas* spp. associated with monocotyledonous species. This suggests a possible interaction between the unknown small molecules synthesized by the NRPS genes and these monocotyledonous plants. *X. albilineans* possesses a copy of the *cbhA* gene, which is present only in xylem-associated xanthomonads including *Xylella fastidiosa* and in *Ralstonia solanacearum*. This gene is absolutely required for spread in the xylem vessel and the action of the encoded enzyme is possibly to degrade the pit membrane between xylem elements. This *X. albilineans* strain also encodes two polygalacturonase genes that might be required in pectin degradation to the same purpose (Pieretti *et al*. 2009). *Xanthomonas albilineans* strain GPE PC73 does not have *gum* genes to produce xanthan gum, which may allow pathogen spread inside the xylem vessel without blockage. This may promote pathogen transmission by infected cuttings, since complete obstruction of the xylem vessels would lead to rapid death of the host (Pieretti *et al*. 2009).

Comparative genome analysis has also shed light on the evolutionary events underpinning the emergence of the three pathotypes (A, A* and A^w^) of *Xcci* and the differences in host range and virulence between them. Pathotype A has the broadest host range in citrus while pathotype A* and A^w^ have a very limited host range and have only been isolated from citrus species key lime and alemow. Gordon and colleagues (2015) conducted a comparative genomic analysis of 43 strains of *Xcci* to reveal only extremely low variation in gene content; most of the variant gene content was linked to gene islands and regions of recombination. These findings indicate the role played by horizontal gene transfer (HGT) and recombination for the gene content evolution of these pathotypes in terms of gain and loss of gene content and mutations (Gordon *et al*. 2015). The *avrGf1* gene and its homolog *avrGf2* act as a host range restriction factor in the pathotype A^w^ but the deletion of the *avrGf1* gene does not give an A-like host range to this pathotype. This indicates that the presence or absence of other factors is necessary to explain the host range of these pathotypes. The *xopAD* gene encoding a T3E is found in both A* and A^w^ where it has undergone pathotype specific recombination. Deletion of *xopAD* in the strain IAPAR 306 (which is a strain A) had no effect on pathogenicity on different citrus hosts (Escalon *et al*. 2013). Since *xopAD* shows unique variations in each pathotype, this gene can be used in future studies to identify pathotype-specific host range.

### Ecological genomics

The major sources of inoculum of black rot, which is caused by *Xcc*, are contaminated seeds or transplants grown from contaminated seeds (Krauthausen *et al*. 2006) and *Xcc*-contaminated debris from the previous crop. Black rot-inducing *Xcc* strains have been isolated from cruciferous weeds (Shepherd's Purse) and contaminated non-cruciferous weeds from black rot infested fields (Krauthausen, Laun and Wohanka 2011). Black rot infested Shepherd's Purse can maintain the pathogenic potential of *Xcc* for over two successive overwinter periods and that indicates the potential to serve as a source of primary inoculum in the spring. In order to manage black rot it is important to perform crop rotation, apply field sanitation immediately after harvesting and use clean seeds and seedlings.

The existence of multiple lineages of *Xanthomonas* in the same host plant has been reported: *Xanthomonas* sp. strain CPBF 424 was isolated from the asymptomatic dormant buds of a diseased walnut tree in Portugal, with typical walnut bacterial blight symptoms (Fernandes *et al*. 2018). According to the multilocus sequence analysis (MLSA) of some of the housekeeping genes, this new strain is located between the non-pathogenic *X. arboricola* and *X. prunicola* clusters. CPBF 424 is divergent from both *X. arboricola* pv. *juglandis* (*Xaj*), the causal agent of walnut bacterial blight, and other *X. arboricola* pathovars. Since pathogenicity tests confirmed that the CFBF 424 strain is pathogenic to walnut trees, studies of this strain may provide new insights into xanthomonad pathoadaptations (Fernandes *et al*. 2018).

Comparative genomics of three *Xanthomonas* pathovars infecting diverse fruit trees has allowed an understanding of their evolutionary origin (Midha and Patil 2014). The maximum likelihood phylogeny indicated a close relationship between *Xanthomonas axonopodis* pv. *citri* (*Xac*) the causal agent of canker in citrus and *X. citri* pv. *mangiferaindicae* (*Xmi*), the causal agent of black spot in mango. Even though these pathogens share common host plants belonging to the order *Sapindales*, several differences were identified between these two strains, indicating the dynamic nature of genome evolution that these host-specific pathovars are undergoing. *Xmi* differs from *Xac* by the presence of a large NRPS/PKS cluster, the absence of CRISPR II, a large deletion in the xanthomonadin cluster and a deletion of the LPS cassette. Interestingly *Xac* and *X*. *arboricola* pv. *punicae* (*Xcp*), which do not belong to the same sublineage have similar LPS cassettes and CRISPR elements. These observations indicate the flexibility of these genomes that underpins their evolution for adaptation into different pathosystems (Midha and Patil 2014).

Comparative genomic analysis of pathogenic and non-pathogenic *Xanthomonas* strains that co-exist in the same host plant can give more information regarding the genomic adaptations of a non-pathogenic strain to a particular environment. A pangenome analysis of *Xanthomonas* strains isolated from the healthy rice seed identified a number of unique genes that are shared by *X. maliensis* (that has been previously reported to have a non-pathogenic lifestyle) and rice pathogenic *Xanthomonas* (Triplett *et al*. 2015). Even though *X. maliensis* lacks the T3SS and its associated effectors which are found in pathogenic *Xanthomonas* spp., it does carry genes for other secretions systems including T1SS, T4SS and T6SS, indicating some features similar to pathogenic *Xanthomonas* spp. Accordingly, *X. maliensis* is phylogenetically more related to pathogenic than to non-pathogenic isolates. These observations suggest *X. maliensis* is a possible intermediate in the evolution of pathogenic from non-pathogenic strains through the acquisition of genes that are usually present in pathogenic isolates (Triplett *et al*. 2015).

### Outbreaks, epidemiology and population structures

Recent advances in high-throughput sequencing with higher speed and output-to-cost ratios have allowed large collections of a single plant pathovar to be sequenced and compared. Bart and colleagues (2012) sequenced 65 *Xam* strains that cause cassava bacterial blight disease. These 65 strains collected from 12 countries representing three continents and spanning 70 years of evolution, can be considered as an exemplar of sampling diverse strain collection across space and time. Among the studied *Xam*, strong clustering by geographic origin was observed (Bart *et al*. 2012). This indicates the significant influence of environmental conditions on pathogen population structure that results in different dominant strains across different geographical regions. Interestingly, no increase in the number of effector genes was observed in strains sampled from the same location during different years. Even though these highly conserved effectors can be subjected to loss or mutation in the face of selection pressure, these can be considered as ideal targets for developing resistance strategies.

In 2012, cotton plants grown in areas centered on Clarksdale, Mississippi were exhibiting symptoms of cotton bacterial blight. The symptoms had been controlled for more than half a century by using resistant germplasm. According to the phylogenic analysis conducted by Phillips *et al*. (2017), the current outbreak was caused by two strains that cluster with race 18 *Xanthomonas citri* pv. *malvacearum* (*Xcm*) strains. The recent outbreak was not due to an introduction of a new *Xcm* race, and also was not due to allelic or expression differences among virulence proteins that could cause re-emergence of cotton bacterial blight. Since the newly isolated *Xcm* was able to trigger the hypersensitive response (HR) on resistant cotton cultivars it was concluded that the recent outbreak of *Xcm* in the USA was due to re-emergence of race 18 clade *Xcm* likely due to cultivation of susceptible cultivars (Phillips *et al*. 2017). Race 18 *Xcm* might have maintained their populations below detection level on alternative hosts, since resistant cottons were planted in 1990s and early 2000s or might have been re-introduced into the southern USA by contaminated seeds.

Recent outbreaks of *Xanthomonas* wilt of enset and banana disease, caused by *Xvm* have been recorded from several major banana-growing countries in East and Central Africa between 2001 and 2008 (Tushemereirwe *et al*. 2004; Ndungo *et al*. 2006). Sequence analysis on 20 isolates collected between 1968 and 2005 from the regions where outbreaks had been recorded revealed only few variations (Aritua *et al*. 2009). Due to this homogenous nature of the *Xvm* populations with respect to time of isolation, geographic origin or host it is believed that the recent outbreak of *Xanthomonas* wilt in the Africa was due to the spread of the *Xvm* that originated in Ethiopia in 1968. Aritua and colleagues were also able to identify a similarity between *Xvm* isolates and the strains of *X. vasicola* from sorghum, maize and sugarcane originating in Africa. This *musacearum* pathovar might have evolved recently to become pathogenic on enset due to the close association with *X. vasicola*-infected sorghum.

Clustered regularly interspaced short palindromic repeats (CRISPRs) and variable number of tandem repeats (VNTRs) have been identified as high-resolution molecular typing tools that can be used to assess evolutionary changes within closely related microbial isolates by epidemiological typing. The CRISPR/cas system and multiple-locus VNTR analysis (MLVA) system is already used in global surveillance of plant pathogens evolution and epidemiological analysis (Rezzonico, Smits and Duffy 2011; Pruvost *et al*. 2014). Both of these genotyping methods have been used to study the new outbreaks of strawberry plant pathogen, *X. fragariae* (Gétaz *et al*. 2018). In this study, 58 bacterial strains of *X. fragariae* with miscellaneous geographic origins and years were analysed and two distinct groups that evolved independently before the first *X. fragariae* isolate was identified.

### Type III effector identification and evolution

Machine learning approach combined with *in planta* translocation assays has been successfully used in recent studies to predict and validate novel T3Es. Previous approaches to predict effectors were limited by the fact that they were based on analysis of single molecular characteristics between effectors and non-effectors. Since a genome-wide machine learning approach scores all the open reading frames (ORFs) based on a large set of features that cover a range of evolutionary, genomics and biochemical characteristics, it can be used to identify T3Es that were missed by previous methods. Using a machine learning approach, Teper *et al*. (2016) identified seven novel T3Es in *X. euvesicatoria* also known as *X*. *campestris* pv. *vesicatoria* (*Xav*). Only three of the identified effectors belong to previously known effector families, while one of the novel effectors (XopAP) was identified as a factor that contributes to the disease development in plants infected with *Xav*.

A machine learning approach has also been used to detect T3E repertoires in 44 *X. arboricola* genomes representing 23 commensal and 21 pathogenic strains (Merda *et al*. 2017). *Xanthomonas arboricola* group A strain CFBP 2528 was used as the reference strain in this study due to the lower number of contigs of that strain. Based on the prediction, seven ancestral core T3E genes were found and the predicted T3E repertoires were highly variable with some strains with no effector and others with up to 34 effectors. The pan T3E effectome of *X. arboricola* comprised 57 predicted T3E. Eleven of the predicted effectors were found in both pathogenic and commensal strains, while 46 other effectors were only in pathogenic strains. About 12 novel T3E genes were identified in *X. arboricola* and six of these showed weak similarity to T3E known in other *Xanthomonas* species (Merda *et al*. 2017).

Draft genome sequences have also been used in genome comparisons and to obtain information on genome contents. Peng *et al*. (2016) obtained a complete genome sequence of *X*. *translucens* pv. *undulosa* (*Xtu*) strain XT4699 using SMRT sequencing technology. This sequence was compared with Illumina draft genome sequences of nineteen *X*. *translucens* strains, which were collected from wheat or barley in different regions and at different times. According to the full genome sequence of XT4699, 8 TAL effector genes that are phylogenetically distinct from other xanthomonad TAL effectors were identified. XT4699-*tal7* was conserved among 8 of 12 of the sequenced *Xtu* strains. From the genomic data of *X*. *translucens* strains, 39 putative T3E genes not including TAL effectors were identified (Peng *et al*. 2016). Genomic comparisons among different pathovars or within pathovars also revealed variations on T3E repertories. It was speculated that these variations might be related to the host range adaptations of these strains.

### Overcoming host resistance and rapid emergence of virulent strains

Rice contains at least 30 bacterial blight resistant genes that have been used in conventional breeding and this has put intense selection pressure on *X. oryzae* strains to overcome existing resistance (Liu *et al*. 2014). Rapid emergence of these strains has been studied extensively in the recent years. India is a major region of diversity of rice as well as its pathogen, *Xoo*. Indian *Xoo* strains belonging to lineage L-I and L-III can overcome resistance mediated by either *xa5* or *xa13* but not both, suggesting that lineage L-I and L-III strains acquired the ability to overcome either of these resistances independently (Midha *et al*. 2017).

### Genome dynamics/plasticity-studying evolutionary processes shaping pathogen populations

Comparisons of the complete genome sequence of *Xoo* strain PXO99A with those of strains MAFF311018 (MAFF) and KACC10331 (KACC), which are highly similar to one another, have provided direct evidence that the *Xoo* genome is highly plastic and rapidly evolving (Salzberg *et al*. 2008). The PXO99A genome encodes19 TAL effectors and has at least 10 major chromosomal rearrangements relative to KACC and MAFF strains, resulting in 29 distinct syntenic blocks. Most of these rearrangements appear to be mediated by a set of transposable elements. PXO99A contains fewer classes of elements, but more copies of ISXo8, IS1114/ISXoo4 and ISXo2 while MAFF and KACC have nearly identical numbers of IS elements. Sequences unique to PXO99^A^ include a 38 kbp region that includes several IS elements and is flanked by direct repeats of ISXo5, indicating that the IsXo5 element was involved in the genomic rearrangement that led either to loss of the locus from MAFF and KACC or gain of the locus in PXO99^A^. The PXO99^A^ strain also has a near-perfect tandem duplication of 212 kb and this repeat is flanked by insertion of ISXo5 at each end and between the two copies. The 212 kb-segment occurs once in the MAFF and KACC sequences. Since these two regions are 100% identical (except for a single base difference in one IS copy), this duplication represents a remarkably recent event. A rapidly evolving CRISPR region contains phage infections unique to the PXO99A lineage and this provides one of the most unique records of differentiation among PXO99^A^, MAFF and KACC. PXO99^A^ has the largest CRISPR region with 75 spacer elements while MAFF and KACC contain just 48 and 59 spacers, respectively. The majority of the spacers are unique to each strain, indicating the rapid evolution of these regions.

*Xanthomonas citri* str. Xc-03–1638-1–1 (Xc-A44) is an A group strain that has two plasmids: a 294kb-copper resistance gene-harbouring plasmid and 99kb-pathogenicity plasmid. The size of the pathogenicity plasmid was approximately equal to the combined size of two plasmids in the *X. citri*. str. 306. Gochez and colleagues sequenced this plasmid using PacBio sequencing to reveal the presence of 4 TALEs. Xc-03–1638-1–1 is the only *X. citri* strain with all four copies of TALEs carried on a single plasmid. Interestingly none of these TALEs were 100% identical to the TALEs from *X. citri* strain LM180, another Cu resistant strain that was isolated simultaneously with Xc-03–1638-1–1 (Gochez *et al*. 2018). The association of Tn*-3* like transposon and of repetitive elements suggests that alternative structures of this pathogenicity plasmid exist in nature.

Recombination-mediated evolution of bacterial plant pathogens is important for the colonisation in novel hosts and new disease emergence. Jibrin *et al*. (2018) compared two newly sequenced Nigerian *Xanthomonas* strains (N1 and N38) with 70 previously published *X. perforans* and *X. euvesicatoria* strains. Recombination was identified as the major driving force of evolution of *X. perforans* and *X. euvesicatoria* strains, with *X. perforans* showing more evidence for recombination than *X. euvesicatoria* (Jibrin *et al*. 2018). The analysis showed that both *X. perforans* and *X. euvesicatoria* have open and highly dynamic pangenomes. Recombination had also affected the *hrp* genes, the *hrp* conserved (*hrc*) genes and *hrp*-associated (*hpa*) genes of *X. euvesicatoria* and *X. perforans* lineages. They observed recombination generated intraspecific variation in T3SS alleles, as indicated by genealogies that are incongruent among T3SS genes and with the core genome tree.

An integrative and conjugative element (ICE) was found in *Xaj* str. CFBP 7179 (*Xaj*-ICE), isolated from vertical oozing canker (VOC) infected walnut trees in France (Cesbron *et al*. 2015). This ICE shows 100% gene similarity to the genomic island (GI) found in bacteria from different genera, specifically *Pseudomonas aeruginosa* strains and *Stenotrophomonas maltophilia* strain D457, which belongs to the Xanthomonadaceae family. The ICE carried genes that are involved in copper resistance (*copA, copB, copC, copD, copF, copG, copK*) and most of these strains were confirmed to be copper resistant. The presence of this ICE in the *Xaj* str. CFBP 7179 might be an indication of the expansion of bacterial genomes due to the extensive use of copper in disease management. Since *Xaj*-ICE is the first known ICE identified in *Xanthomonas*, it might have been acquired by lateral transmission from a strain belonging to a different genus (Cesbron *et al*. 2015).

## XANTHOMONAS FACTORS CONTRIBUTING TO DISEASE

The determination of the genome sequence of several species and pathovars of *Xanthomonas* has facilitated functional analyses aimed at understanding the molecular basis of virulence and adaptation. These functional studies have utilized different infection models and inoculation techniques (spraying, leaf clipping, mesophyll infiltration and vascular inoculation) to identify factors that play a significant role in the different phases of the disease cycle. In the following section, we will discuss recent advances in the study of virulence of *Xcc* and drawn parallels with other *Xanthomonas* spp. to demonstrate the adaptable and environmentally responsive nature of these pathogens during infection. In particular, we will highlight cell–cell signalling, second messenger signalling, two component systems, TonB-dependent outer membrane transporters and type III-secreted effectors (Table S2, Supporting Information).

### Cell–cell signalling and associated pathways

The synthesis of particular virulence factors in *Xanthomonas* spp. is controlled by cell–cell signalling (quorum sensing) mediated by molecules of the DSF family, which are *cis*-2-unsaturated fatty acids (Table S2, Supporting Information) (Ryan *et al*. 2015; Zhou *et al*. 2017).

The predominant DSF family signals in *Xcc* are *cis*-11-methyl-2-dodecenoic acid (called DSF) and *cis*-2-dodecenoic acid (BDSF) (Ryan *et al*. 2015; Zhou *et al*. 2017). Synthesis and perception of the DSF signal require products of the *rpf* gene cluster. The synthesis of DSF is dependent on RpfF, a member of the crotonase superfamily with both desaturase and thioesterase activity that generates the signal molecule from a hydroxylated fatty-acyl ACP. A two-component system comprising the sensor kinase RpfC and regulator RpfG is implicated in DSF perception. Recent work has implicated residues in the extreme N-terminal of RpfC, which is located in the periplasm, in the recognition of DSF, although it is likely that other residues in the membrane-associated sensory input domain are also involved (Cai *et al*. 2017). Signal binding is believed to activate autophosphorylation of RpfC and phosphotranfer to RpfG, a two-component response regulator with an HD-GYP cyclic phosphodiesterase domain; phosphorylation of RpfG activates the protein for cyclic di-GMP degradation, which affects a range of cellular processes (discussed below).

In *Xcc*, the *rpfF* gene is linked to *rpfB* that encodes a fatty acid CoA ligase; *rpfB* is expressed as an operon with *rpfF*, although *rpfF* also has its own promoter. RpfB has been proposed to have a role in the mobilization of saturated free fatty acids generated by the thioesterase action of RpfF, allowing these free fatty acid derivatives to be used in phospholipid biosynthesis (Hu *et al*. 2018). However, more recent work has shown that *rpfB* mutants have elevated levels of DSF family signals, leading to the suggestion that RpfB is involved in signal degradation (Hu *et al*. 2018). This is intriguing since isolated RpfB has no apparent ligase activity against BDSF *in vitro*.

Comparative transcriptome analysis of wild type and *rpfF*, *rpfC* and *rpfG* mutants has suggested additional complexities in DSF signal perception and transduction (An *et al*. 2013a, 2014). Specifically, the effects of these mutations suggested alternative sensors for DSF and alternative regulators that interact with RpfC rather than a direct pathway (An *et al*. 2013a, 2014). Although alternative regulators that interact with RpfC have yet to be defined XC_2579 (RpfS) was identified as a second sensor for DSF in *Xcc* (An *et al*. 2013a, 2014). RpfS is a histidine sensor kinase containing an N-terminal PAS domain (PAS_4 domain). This PAS domain binds DSF and is required for regulation. Within the genes controlled by DSF, RpfS controls expression of a subset of genes and functions during epiphytic of *Xcc*.

DSF signalling has also been implicated in facilitating the entry of *Xanthomonas* spp. into plants, controlling synthesis of a factor that acts to reverse stomatal closure in *Arabidopsis* (Gustavo *et al*. 2009; Kakkar *et al*. 2015). This plant response is part of the innate immune system and can be induced by bacteria, bacterial components and the plant hormone abscisic acid. The *Xanthomonas* spp. factor responsible has not been identified but can be extracted from culture supernatants with ethyl acetate. The plant target is equally unknown but may be a signalling pathway involving the guard cell-specific *Arabidopsis* Mitogen-Activated Protein Kinase3 (Gustavo *et al*. 2009; Kakkar *et al*. 2015). Whether this *Xcc* factor similarly affects the pores of the hydathodes is not known. Although these pores resemble stomata, they do not respond to established elicitors of plant innate immunity (Melotto, Underwood and He 2008). Although the ethyl acetate extract would also contain DSF and derivatives, genetic evidence suggests that it is not the DSF signal molecule *per se* that acts to reverse stomatal closure. Furthermore, DSF acts to induce plant defences in the leaves of *Arabidopsis*, *Nicotiana benthamiana* and rice (Gustavo *et al*. 2009; Kakkar *et al*. 2015).

*Xanthomonas* spp. synthesises a second diffusible factor called DF, whose synthesis depends upon XanB2, which is encoded within the *pig* locus that directs synthesis of the characteristic yellow pigment xanthomonadin (Zhou *et al*. 2013a, 2013b). DF is associated with regulation of xanthomonadin biosynthesis, cell viability, epiphytic colonization and systemic invasion (Zhou *et al*. 2013a, 2013b). XanB2 is a unique bifunctional enzyme with three putative domains that hydrolyzes chorismate to produce 3-hydroxybenzoic acid (3-HBA) and 4-hydroxybenzoic acid (4-HBA). 3-HBA is associated with the xanthomonadin biosynthesis whereas 4-HBA is associated with CoQ8 biosynthesis and antioxidant activity. DF could be considered a metabolite cue rather than a genuine cell–cell signal molecule, as 3-HBA can be incorporated into xanthomonadin. Nevertheless, there is an increasing appreciation of the importance of transfer of metabolites between cells particularly within bacterial communities.

### Intracellular signalling mediated by nucleotide second messengers

To date, a regulatory role for the nucleotide intracellular signals cyclic di-GMP (c-di-GMP) and cyclic GMP (cGMP) has been described for *Xanthomonas* spp. (Table S2, Supporting Informtion) (Ryan 2013; An, Tang and Dow 2017). The cellular level of these signals results from a balance between synthesis and degradation. For c-di-GMP three protein domains are implicated in these processes: the GGDEF domain catalyses synthesis of c-di-GMP from 2 molecules of GTP whereas EAL and HD-GYP domains catalyse hydrolysis of c-di-GMP, first to the linear nucleotide pGpG and then at different rates to GMP (recently reviewed by Dahlstrom and O'Toole 2017; Jenal, Reinders and Lori 2017). The *Xcc* 8004 genome encodes 37 proteins with GGDEF, EAL or HD-GYP domains. Although many of these are conserved in other *Xanthomonas* spp., the number of proteins progressively decreases from *Xcc* to *Xca* through to *X*. oryzae pv. *oryzicola* (*Xoc*) and *Xoo*. The effects of deletion/mutation of the genes encoding some of these proteins have been reported to effect virulence and biofilm formation (Wei *et al*. 2016; Yang *et al*. 2016; Su *et al*. 2016b; Li *et al*. 2018; Xue *et al*. 2018). The majority of these proteins contain further domains that have a role in recognition of different environmental cues or comprise regulatory elements of two-component systems, such as the HD-GYP domain protein RpfG introduced above. A comprehensive mutational analysis has shown a role for a number of these proteins in virulence, as measured by leaf clipping, and in virulence factor production (Ryan *et al*. 2007; An, Tang and Dow 2017). A body of work has now identified a role for c-di-GMP in regulation of a wide range of functions in *Xcc* that include adhesion, biofilm formation, motility, synthesis of polysaccharides and synthesis of extracellular enzymes (Ryan *et al*. 2007; An, Tang and Dow 2017). The outcome of these experiments suggest that a number of c-di-GMP signalling proteins are organized as a network that allows integration of information from diverse environmental inputs to modulate particular functions such as extracellular enzyme synthesis. In contrast, other c-di-GMP signalling systems appear to be dedicated to alternative specific tasks. Whether different subsets of proteins are important in different phases of the disease cycle remains to be determined.

C-di-GMP exerts a regulatory action by binding to a range of receptors or effectors, such as small ‘adaptor’ proteins with a PilZ domain, transcription factors and riboswitches (Chin *et al*. 2010; 2012; Guzzo *et al*. 2013). The cyclic AMP receptor-like protein Clp links Rpf/DSF signalling (and alteration in c-di-GMP) to the expression of genes encoding extracellular enzymes. C-di-GMP binding to Clp prevents the binding of the transcriptional activator to the promoters of several genes encoding extracellular enzymes (Chin *et al*. 2010, 2012; Guzzo *et al*. 2013). Lowering of the c-di-GMP levels, for example, by activation of DSF cell–cell signalling, reverses this effect. Clp also regulates biofilm dynamics in response to changes of the level of c-di-GMP. Mutation of *clp* causes downregulation of *manA* that encodes an endomannanase involved in biofilm dispersal and upregulation of *xag* genes, which synthesize a polysaccharide associated with biofilm formation. The nature of this polymer remains to be established.

All *Xanthomonas* genomes encode four proteins that have a PilZ domain (Yang *et al*. 2015). In *Xcc* strain 8004 these are designated XC_0965, XC_2249, XC_2317 and XC_3221 (Chin *et al*. 2010, 2012; Guzzo *et al*. 2013). Mutational analysis indicates that XC_0965, XC_2249 and XC_3221 make a significant contribution to the virulence in Chinese radish. A few details of the regulatory action of these proteins (or their orthologs in other *Xanthomonas* spp.) have emerged (Chin *et al*. 2010, 2012; Guzzo *et al*. 2013). XC_0965 and XC_2249 have canonical PilZ domains and hence are predicted to bind c-di-GMP. Both of these proteins positively regulate extracellular enzyme production, whereas XC_2249 additionally affects bacterial motility. In contrast, XC_3221 has a non-canonical type II PilZ domain, does not effectively bind c-di-GMP, but does influence pilus-dependent motility. XC_3221 exerts this effect by interaction with an enzymatically inactive GGDEF-EAL domain protein, called FimX (XC_1824 in *Xcc* strain 8004), and the PilB pilus motor protein. Although FimX is enzymatically inactive, it can bind c-di-GMP via the EAL domain and then forms a complex with XC_3221. This complex interacts with PilB to influence pilus biogenesis. XC_3221 probably has other cellular functions that contribute to virulence since mutation of *pilA*, which abolishes motility, has a lower effect on virulence than mutation of *XC_3221*. Recently, a new class of c-di-GMP effector of the YajQ family has been identified in *Xcc* (An *et al*. 2013b; Zhao *et al*. 2016). This protein (XC_3703) acts to influence the transcription of genes that contribute to virulence in plants and biofilm formation. The available evidence suggests that XC_3703 exerts its action through protein–protein interactions with the LysR family regulator XC_2801, an interaction that is negatively regulated by c-di-GMP (An *et al*. 2013b; Zhao *et al*. 2016). Comparative transcriptome profiling indicates that YajQ is involved in further regulatory pathways not involving XC_2801, but these are currently undefined. *In silico* analysis has predicted riboswitch candidates in *Xcc* that potentially bind c-di-GMP suggesting that further classes of c-di-GMP effector remain to be discovered (Alkhateeb *et al*. 2016).

Evidence has recently been provided for a cGMP-mediated pathway in *Xcc* that acts in the regulation of virulence, biofilm formation and the transcription of specific virulence genes (An *et al*. 2013b; Ryu *et al*. 2015). This study is one of the first descriptions of the role of cGMP in bacterial biofilm formation and virulence of a bacterial pathogen. The synthesis of cGMP in *Xcc* depends upon XC_0250, which is a guanylate cyclase with a class III nucleotidyl cyclase domain attached to a domain of uncharacterized function with tetratricopeptide repeats. The action of cGMP in *Xcc* depends in part on XC_0249, which has a cGMP-binding domain attached to a GGDEF domain active in c-di-GMP synthesis (An *et al*. 2013b; Ryu *et al*. 2015).

### Environmental sensing (other than cell–cell signalling) and the role of two-component systems and TonB-dependent outer membrane transporters

**Figure 3.**
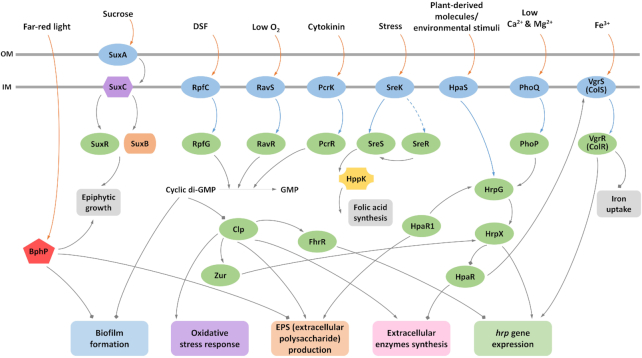
*Xanthomonas* employs multiple systems to link sensing of diverse environmental signals to regulation of appropriate responses. The *Xcc* genome encodes a number of two-component and other systems thought to be involved in environmental sensing and regulation. In a relatively small number of cases the signals that activate these pathways have been established, to include the DSF cell–cell signal, oxygen tension, iron and other metal ions, cytokinin and other plant-derived molecules to include sucrose and light. In addition to two-component regulators, a number of transcription factors have been implicated in downstream signalling pathways that lead to activation of functions associated with virulence and other environmental adaptations. These include the cyclic di-GMP responsive Clp, the type III secretion regulators Zur, FhrR PhoP, HpaR, HrpX, HrpG and HpaR1. Interestingly, VgrR although be shown to be involved in iron uptake also contributes to type III secretion regulation. Code Blue sensor proteins, Green regulatory proteins

Several systematic studies have examined the role of sensing systems in *Xanthomonas*, in particular in *Xcc* that respond to environmental or cellular stimuli in the control of virulence, cellular behavior and gene expression (Table S2, Supporting Information; Fig. 3) (Li *et al*. 2014; Cui *et al*. 2018). The *Xcc* genome encodes a number of two component systems including RpfC/RpfG discussed above. Several of these systems have been implicated in regulation of virulence factor synthesis, biofilm formation or resistance to oxidative stress (Li *et al*. 2014; Cui *et al*. 2018). In some cases, the nature of the signal or cue to which the system responds has been determined (Table S2, Supporting Information; Fig. 3). A key pathway is that involving the transcriptional regulator HrpG, which directly regulates the expression of HrpX, an AraC-type transcriptional regulator controlling expression of the T3SS (Li *et al*. 2014; Cui *et al*. 2018). HrpG is an orphan regulator, as *hrpG* has no linked gene encoding a sensor. Mutational analysis and protein–protein interaction studies have identified HpaS as a putative sensor (Li *et al*. 2014). It is proposed that HpaS responds to plant-derived molecules and other environmental stimuli. HpaS may interact with further regulators, in particular HpaR2, which is encoded by a linked gene (Li *et al*. 2014).

Recent work has also established that light is an important environmental cue influencing *Xcc* interaction with host plants (Bonomi *et al*. 2016; Otero *et al*. 2016). *Xcc* produces a bathy-type photoreceptor called XccBphP that photoconverts between red-absorbing (Pr) and far-red-absorbing (Pfr) states. Upon sensing light, the XccBphP elicits a transcriptional response that leads to down-regulation of xanthan production and biofilm formation and reduced virulence. It has been proposed that XccBphP acts to co-ordinate enhanced virulence factor synthesis to conditions of low light under which the plant tissues exhibit increased susceptibility (Bonomi *et al*. 2016).

*Xanthomonas* spp. have much larger numbers of TonB-dependent receptors (TBDRs) than many eubacteria, as determined by genome analysis (Blanvillain *et al*. 2007; Déjean *et al*. 2013). These proteins are present in the outer membrane of Gram-negative bacteria and transport iron-siderophore complexes and vitamin B12 into the periplasm. The inner membrane energy-coupling TonB-ExbB-ExbD protein complex provides the energy required for transport. Recent evidence has suggested that some *Xcc* TBDRs might transport plant-derived molecules and may have roles in bacterial virulence or survival (Blanvillain *et al*. 2007; Déjean *et al*. 2013). This action was revealed by the definition of specific CUT systems (Carbohydrate Utilization systems containing TonB-dependent transporters) that scavenge plant carbohydrates. One CUT system using *N*-acetylglucosamine from plant origin has been implicated in virulence whereas a second system involved in the mobilization of xylan contributes to phyllosphere fitness (Blanvillain *et al*. 2007; Déjean *et al*. 2013; Boulanger *et al*. 2014).

Most sequenced *Xanthomonas* genomes encode multiple ORFs characterized as σ factor. In *Xcc*, there are 15 such ORFs, of which 10 belong to the extracytoplasmic function (ECF) subfamily, which act under stress conditions to co-ordinate expression of specific genes (Cheng *et al*. 2008; Bordes *et al*. 2011). Recent work has shown that these ECF factors are required for the survival of *Xcc* under stress and also mediate the synthesis of virulence factors. RpoE1 is the best-described ECF σ factor in *Xcc*, where it plays a role in the heat shock response, adaptation to membrane-perturbing stresses, stationary-phase survival, resistance to cadmium and T3SS expression (Cheng *et al*. 2008; Bordes *et al*. 2011b; Yang *et al*. 2018). Currently little or nothing is known of the mechanisms by which these different ECF σ factors are activated or of any regulatory interplay with other systems involved in sensing. It is clear, however, that the array of such sensing systems allows *Xcc* to monitor many aspects of the bacterial environment and to respond in an appropriate fashion.

### Type III effector biology and action

Xanthomonad repertoires of T3Es are diverse and aid virulence and possibly host specificity (Table S1, Supporting Information). This aspect of Xanthomonad biology has been reviewed extensively recently so we just summary key findings (please see Jacques *et al*. 2016). To date, a total of 52 effector families have been identified along with three harpin proteins, which are helper or accessory proteins that assist in effector translocation (www.xanthomonas.org).

The majority of sequenced *Xcc* genomes contain a core set of nine effector genes (*xopR, avrBs2, xopK, xopL, xopN, xopP, xopQ, xopX, xopZ*) (Table S1, Supporting Information). However, considerable variation in effector complement is seen between *Xcc* strains and between *Xcc* and other *X. campestris* pathovars attacking brassicas. Mutation of genes encoding some core effectors leads to reduced virulence and fitness of *Xcc* but this can depend on the strain. It has been suggested that there may be substantial functional redundancy among *Xcc* effectors.

*Xcc* appears to econtain several effector genes that are only found associated with this species: *xopAL1, xopAC, xopAD, xopAH* and *xopAL2* are unique to *Xcc* strains. Whether these genes affect host specificity requires further functional testing. Furthermore, *Xcc* strains appear to encode a specific set of transcription activator-like (TAL) effectors when compared to other *Xanthomonas* spp. and pathovars (Table S1, Supporting Information) (Denancé *et al*. 2018). These effectors are considered intriguing candidates for determinants of host specificity and tissue preference.

Effectors from several *Xcc* strains do affect restriction of host range. Avirulence factors typically determine specificity at the pathogen race/host cultivar level by triggering defence responses in hosts with a corresponding specific resistance gene. AvrXccC and AvrXccE1 determine host specificity for *Xcc* on mustard and Chinese cabbage, respectively, and AvrBs1 determines non-host resistance on pepper ECW10R (He *et al*. 2007).

Effectors may also determine tissue specificity: AvrAC has been shown to trigger resistance specifically in the vascular system of *Arabidopsis thaliana* Col-0 but has no discernible effect on growth in mesophyll tissue (Xu *et al*. 2008). It is not clear whether this reflects the tissue-specific expression of the corresponding resistance gene. The method of inoculation used in these studies bypasses the hydathodes, which are the usual route of entry into the vascular system and have long been suggested to contribute to resistance. Recent work has shown that bacteria enter the hydathode without apparent restriction but subsequently activate plant immune responses that limit growth in the epithem (Cerutti *et al*. 2017). Suppression of these defence responses, which is required to allow disease, depends on the type III secretion system and associated effectors. Nevertheless, *avrAC* acts to restrict pathogen growth upon hydathode infection in *Arabidopsis* Col-0, consistent with the earlier study (Cerutti *et al*. 2017). Identification of conserved effector or avirulence genes is a major step towards the cloning of the matching resistance genes, which may be found not only in brassicas but also in non-host plants, and their subsequent deployment in agriculture.

## XANTHOMONAS EPS AND LPS

### The EPS xanthan

**Figure 4.**
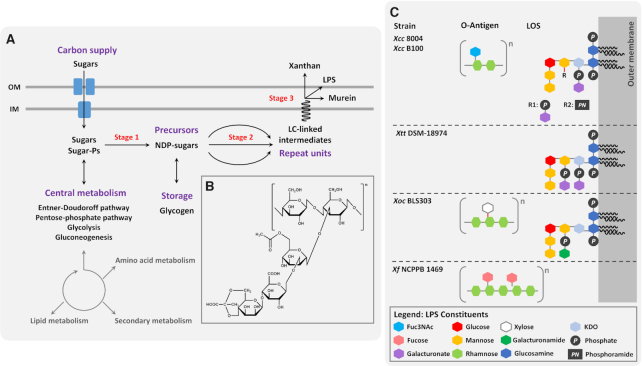
Polysaccharide biosynthesis and selected lipopolysaccharide structures in xanthomonads. **(A)**, An overview indicates how the biosynthesis of polysaccharides and glycoconjugates is embedded in the *Xanthomonas* metabolism. Main stages of polysaccharide biosynthesis are marked in red. **(B)**, In an inlay, the structure of a pentasaccharide repeat unit of the exopolysaccharide xanthan is given. Two glucose residues contribute to the xanthan main chain, with a trisaccharide side chain attached to every second glucose residue. The mannose residues of the side chains can be modified at varying degrees. The proxymal mannose close to the main chain is depicted with an acetyl group, while the terminal mannose carries a pyruvyl group. Alternatively, this mannose can be acetylated. **(C)**, LPS structures are indicated for *X. campestris* pv. *campestris* 8004, which have been partly confirmed for strain B100, for *X. translucens* pv. *translucens* DSM-18 974, for *X. oryzae* pv. *oryzicola* BLS303, and for *X. fragariae* NCPPB 1469. Main constituents are identified in the graphical legend. In several cases, side chains are linked non-stoichiometrically. Such non-stoichiometric linkages are marked in red. For *X. fragariae* NCPPB 1469, part of the monosaccharide side chains were linked in irregular intervals to the main chain.

The EPS xanthan is a characteristic component of all xanthomonads with the exception of those with reduced genomes where the xanthan biosynthetic genes (the *gum* cluster) have been lost (Becker and Vorhölter 2009; Pieretti *et al*. 2009). The polysaccharide encloses the outer membrane, to which it is not covalently linked. (Becker and Vorhölter 2009). Xanthan has a cellulose-like (1→4)-*β*-D-glucose polymer backbone, where every second glucose residue of the main chain carries a (1→3)-linked trisaccharide side chain of *β*-d-mannose-(1→4)-*β*-D-glucuronic acid-(1→2)-α-D-mannose (Fig. 4). While this carbohydrate structure is highly conserved, there are additional non-sugar decorations, with the terminal mannose residues being acetylated or pyruvylated at varying degrees while mannose residues proximal to the main chain are acetylated, again to varying extents.

The role of xanthan in pathogenesis may be complex and vary among *Xanthomonas* strains and host plants. *Xcc* mutant strains deficient in xanthan biosynthesis have reduced aggressiveness against host plants (Katzen *et al*. 1998). Xanthan has been described to suppress induced innate immunity by calcium chelation (Aslam *et al*. 2008) and to induce plant susceptibility to *Xcc* by suppressing callose deposition (Yun 2006). For *Xac* xanthan was reported as not essential for pathogenicity in citrus canker but to contribute to epiphytic survival (Dunger *et al*. 2007) while in another experiment a xanthan-deficient mutant was reduced in growth and survival on leaf surfaces and provoked reduced disease symptoms (Rigano *et al*. 2007a). Xanthan pyruvylation has an influence on the virulence of *Xcc* (Bianco *et al*. 2016). For *Xoo* the O-antigen component of LPS was shown to induce rice defence responses, which were suppressed by xanthan, resulting in a modulation of the defence response of the host plant (Girija *et al*. 2017). Xanthan is also a key component of the biofilm matrix in *Xanthomonas* (Dow *et al*. 2003). Besides its pathogenicity-related features, xanthan is relevant as a commercially produced thickening agent. Xanthan is commercially produced by fermentation in large scale (García-Ochoa *et al*. 2000) and employed in industry, in particular as a component of food, cleansing materials, pharmaceutics and in drilling for oil and natural gas.

Xanthan biosynthesis originates from the interconvertible metabolic intermediates glucose 6-phosphate and fructose 6-phosphate, which are linked to UTP and GTP to obtain the nucleotide sugars UDP-glucose, UDP-glucuronic acid and GDP-mannose in the first main step of xanthan biosynthesis. The sugar residues of these compounds are transferred to a polyprenol lipid carrier (LC, most likely undecaprenol diphosphate), thereby forming a lipid-linked linear glucose-glucose-mannose-glucuronic acid-mannose pentasaccharide in the second main step of xanthan biosynthesis. This pentasaccharide reflects the repeat unit of the xanthan molecules. Consistent with the Wzy-dependent model for heteropolysaccharide biosynthesis of Enterobacteriaceae (Raetz and Whitfield 2002; Whitfield 2006), the LC-linked pentasaccharide is assumed to be subsequently flipped to the outer surface of the inner membrane, where it is polymerized and exported via a specific channel in the outer membrane (Vorhölter *et al*. 2008). Pyruvylation and acetylation reactions of the mannose residues occur at the LC-linked pentasaccharides, but the exact sequence of the reactions is unclear. Acetyl and pyruvyl groups are donated from the central metablic intermediats acetyl-coenzyme A and phosphoenolpyruvate, respectively.

While genes for the synthesis of the xanthan precursors UDP-glucose and GDP-mannose, which are likely to be also LPS precursors (Vorhölter, Niehaus and Pühler 2001), are clustered with additional LPS biosynthetic genes (Vorhölter, Niehaus and Pühler 2001), the genes that code for the generation of lipid-linked intermediates and the subsequent polymerisation and export of xanthan form a cluster of 12 successive genes with an operon-like identical direction of transcription, *gumB*to *gumM*. Mutagenesis has revealed the functions of most of the *gum* gene products, identifying GumD, GumM, GumH, GumK and GumI as glycosyltransferases that build the pentamer repeating units of xanthan at the LC, and GumF, GumG and GumL as acetyltransferases and pyruvyltransferase, respectively. Similarities to model proteins from Enterobacteriacea paved the way for a model that suggested GumJ as a flippase, which transferred the LC-linked pentasaccharides to the periplasmic face of the inner membrane where it may be polymerized by GumE. The first two genes of the *gum* cluster, *gumB* and *gumC*, are likely to encode components of a channel similar to the Wza/Wzc complex (Collins *et al*. 2007) of *E. coli* that spans the outer membrane and the periplasm and facilitates xanthan export. These GumB and GumC proteins (Jacobs *et al*. 2012; Galván *et al*. 2013; Bianco *et al*. 2014) and two of the *gum* gene products coding for glycosyltransferases, *gumI* (Salinas *et al*. 2011) and *gumK* (Barreras *et al*. 2008; Salinas *et al*. 2016), have been characterised in more detail over the past years. Unfortunately, there is little experimental evidence available regarding the putative flippase GumJ and the suggested polymerase GumE.

The phosphorylated sugars that are requisitioned to synthesise cell surface constituents such as EPS and LPS can either be imported, obtained from glycogen breakdown, or be synthesised from citrate cycle intermediates by means of gluconeogenesis (Tang *et al*. 2005). Xanthomonads have an astonishing repertoire of sugar importers in their inner membrane (Vorhölter *et al*. 2008), plus a vast variety of outer membrane importers, in particular TonB-dependent receptors (Blanvillain *et al*. 2007; Serrania *et al*. 2008; Déjean *et al*. 2013; Boulanger *et al*. 2014) that may also be involved in trans-envelope signalling (Wiggerich and Pühler 2000; Vorhölter *et al*. 2012). The phosphorylated sugars used to synthesize EPS and LPS are also intermediates in pathways leading to the synthesis of glycogen, in catabolism to obtain energy or as building blocks that are ultimately utilised to generate biomass. Xanthomonads mainly employ the Entner–Doudoroff pathway and to a lesser extent the pentose-phosphate pathway for the catabolic utilisation of such intermediates (Schatschneider *et al*. 2014). The Embden–Meyerhof–Parnas pathway (for glycolysis) was recently revealed as an additional catabolic route in xanthomonads (Schatschneider *et al*. 2013). This pathway involves a non-canonical pyrophosphate-dependent phosphofructokinase (Frese *et al*. 2014). Flux analysis produced clear evidence for a remarkable flexibility of the central metabolism, where a major redistribution of resources from xanthan to biomass can occur (Schatschneider *et al*. 2013). However, only minimal flux was observed for glycolysis under standard laboratory growth conditions (Schatschneider *et al*. 2014).

### Xanthomonas LPS

LPS are main components of the outer leaflet of the outer membrane. Usually, LPS are tripartite glycoconjugates with a glycolipid part called lipid A that carries a core oligosaccharide, and a polysaccharide termed O-specific antigen or O-antigen. LPS that lack the O-antigen component are called lipooligosaccharide (LOS) or rough-type LPS. LPSs play a vital role for bacterial growth, providing a barrier to antimicrobial compounds and protection against stresses as well as being important for the structural properties of the outer membrane. In addition, LPSs have an involvement in adhesion and host recognition (Silipo *et al*. 2010). LPS from different *Xanthomonas* species have been shown to induce defence-related responses in both host and non-host plants (Meyer, Pühler and Niehaus 2001; Newman *et al*. 2002). The acylation and phosphorylation pattern of *X*. *campestris* lipid A influences its ability to trigger the innate immune response of its host plant *Arabidopsis thaliana* (Silipo *et al*. 2008). The *Xoc* O-antigen was required for full virulence and type III protein secretion (Wang, Vinogradov and Bogdanove 2013) while the O-antigen component of *Xoo* LPS-induced rice defence responses during infection which were suppressed by xanthan (Girija *et al*. 2017).

While lipid A is rather conserved among many Gram-negative bacteria, there is more variation among core oligosaccharides and O-antigen structures may vary even within an individual *Xanthomonas* spp. Only a limited number of LPS structures has been determined for xanthomonads so far. A selection of these structures is displayed in Fig. 4. For *Xcc*, the structure of the O-antigen repeating unit (Molinaro *et al*. 2003) as well as the LOS structure (Silipo *et al*. 2005) have been determined for the strains 8004 and B100 (Braun *et al*. 2005; Kaczyński *et al*. 2007). Attached to the acylated diglucosamine of the lipid A, the *Xcc* 8004 core oligosaccharide consists of an invariable 3-deoxy-D-manno-oct-2-ulosonic acid (KDO)-mannose-glucose trisaccharide that carries at the KDO a galacturonyl residue that is linked via a phosphate group. An additional galacturonyl phosphate is linked to the mannose residue, which can alternatively be substituted by a phosphoramide group. Finally, the glucose can non-stoichiometrically carry a mannose disaccharide. The complete LOS structure is also available for the *X*. *translucens* pv. *translucens* (*Xtt*) type strain (Steffens *et al*. 2017) while its total LPS monosaccharide composition indicates rhamnose and xylose as O-antigen constituents. The LOS of *Xoo* was found to share the core structure with *Xcc* (di Lorenzo *et al*. 2016), but the authors do not mention the phosphoramide found in *Xcc* LOS. In the LOS of *Xoc*, no galacturonylphosphate side chain was attached to the KDO, while a galacturonamide was linked via a phosphate group to the mannose residue adjacent to the KDO (Wang, Vinogradov and Bogdanove 2013). While all these core structures agree largely, longer core oligosaccharides have recently been determined for *Xac* strain Xcc99–1330, where the LOS was characterised as an acylated diglucosamine with an oligosaccharide that consists of two KDO, three galacturonic acids, five hexoses, four rhamnoses and one Fuc3NAc residue, bearing two phosphate groups while to O-antigen was a rhamnose polymer (Casabuono *et al*. 2011). In addition, some lipid A structures are available (Silipo *et al*. 2004). Exact O-antigen repeating unit structures were elucidated for the *Xcci* strains NCPPB 3562 and CFBP 2911 (di Lorenzo *et al*. 2017), for *X. oryzae* pv. *oryzycola* BLS303 (Wang, Vinogradov and Bogdanove 2013) as well as for several other xanthomonads (Molinaro *et al*. 2000a, 2000b, 2001). A common theme of *Xanthomonas* O-antigen repeat units appears to be a polyrhamnan main chain with monosaccharide side chains in many strains that are in some cases attached to the main chain in an irregular pattern.

Although the mechanism of LPS biosynthesis is particularly well understood for Enterobacteriaceae (Raetz and Whitfield 2002; Whitfield and Trent 2014), the data available for xanthomonads is as yet highly fragmentary. Nevertheless, the available information indicates that concepts accepted for Enterobactericaeae are by and large valid for LPS biosynthesis in xanthomonads.

NDP-sugar precursors are provided in stage 1 of the LPS biosynthesis. The *rmlABCD* genes have been identified to code for dTDP-L-rhamnose (Vorhölter, Niehaus and Pühler 2001). This requires glucose 1-phosphate as starting material, which is generated using the *xanA* gene product, and thereby linked to the synthesis of xanthan. In mutant strains affected in *rmlA* and *rmlD*, the rhamnose monosaccharides obtained by acid hydrolysis of LPS were reduced by more than 90%. DNA sequence similarity pointed to two additional genes, *gmd* and *rmd*, that were annotated to encode the synthesis of GDP-D-rhamnose from the xanthan precursor GDP-mannose (Vorhölter, Niehaus and Pühler 2001). This second rhamnose source might explain the remaining quantity of rhamnose in the *rmlA* and *rmlD* mutants. The biosynthetic pathway of dTDP-3-acetamido-3-deoxyfucose was reconstructed based on DNA sequence analysis; this pathway originates from a dTDP-L-rhamnose precursor (Vorhölter, Niehaus and Pühler 2001). This novel pathway was subsequently confirmed experimentally for similar gene products of *Aneurinibacillus thermoaerophilus* (Pfoestl *et al*. 2003). In addition, there is biochemical evidence for the epimerisation of UDP-glucuronic acid to UDP-galacturonic acid, while genome data revealed the gene for this enzymatic conversion (Vorhölter *et al*. 2008). Genome analysis revealed also the genes that drive the synthesis of UDP-*N*-acetylglucosamine (UDP-GlcNAc) and CMP-KDO (Vorhölter *et al*. 2008), probably precursors of the lipid A and LPS core of xanthomonads, respectively.

As with other Gammaproteobacteria, the lipid A and the LPS core are expected to be synthesized at the inner face of the inner membrane to obtain LOS molecules. The LOS molecules are then exported to the outer face of the inner membrane by means of a specific ABC family exporter termed MsbA (Raetz and Whitfield 2002). Unfortunately, there is little available evidence regarding the biosynthesis of these structures in xanthomonads. However, in *X*. *campestris* a distinct LC-linked galacturonide was detected, with UDP-galacturonic acid as a likely precursor (Baldessari, Ielpi and Dankert 1990). It is tempting to speculate that the galacturonyl phosphate residues of the LPS core are transferred in a distinct biosynthetic step from these LC-linked intermediates by a mechanism that resembles the lipid A modification in *E. coli* where the unusual monosaccharide 4-amino-4-deoxy-L-arabinose is added (Raetz *et al*. 2007).

Subsequently, the O-antigen part of the LPS is expected to be added to the LOS by means of an enzyme termed LPS ligase. As a prerequisite, mature O-antigen is required at the outer face of the inner membrane. The O-antigen is synthesised and exported beforehand in a separate process. The architecture of a channel-forming O-antigen exporter has been determined recently, pointing to a processive O-antigen translocation mechanism (Bi *et al*. 2018). Based on a first approximation of genome data, the Wzm/Wzt ABC-transporter model developed for Enterobacteriaceae may fundamentally describe O-antigen biosynthesis and export in xanthomonads (Vorhölter, Niehaus and Pühler 2001), where functions important for LPS biosynthesis were related to the *wxc* gene cluster. LPS biosynthetic gene clusters were also found in other xanthomonads in a conserved genomic location flanked by the *etfAB* and *metB* genes (Patil and Sonti 2004). However, the gene content of different strains was quite different, with only the *wzm* and *wzt* genes being conserved (Lu *et al*. 2008). In the immediate genomic vicinity of the *Xcc wxc* gene cluster are the *rmlABCD* genes that code for enzymes required to synthesize the LPS precursor dTDP-L-rhamnose, the *xanA* and *xanB* genes that code for NDP-sugar precursors of the EPS xanthan which are also LPS precursors (Vorhölter, Niehaus and Pühler 2001), and the *lpsI* and *lpsJ* genes that were later renamed as *wxcI* and *wxcJ* and that code for 3-oxoacid CoA-transferase subunits which are involved in LPS biosynthesis.

Three distinct regions were distinguished in the *Xcc wxc* gene cluster (Vorhölter, Niehaus and Pühler 2001). While gene products encoded in region 2 were similar to NDP-sugar biosynthetic enzymes, genes from region 1 coded for a Wzm/Wzt ABC-exporter in addition to glycosyltransferases and proteins of unclear function. Mutant strains deficient in these genes were affected in O-antigen biosynthesis (Hötte *et al*. 1990; Vorhölter, Niehaus and Pühler 2001). The functions of the products of some other genes in region 2, for example WxcB, are poorly understood. In *X*. *euvesicatoria*, a *wxcB* knock-out mutant had reduced virulence while effects on the LPS were minimal (Park, Jung and Han 2014). In contrast in an *Xcc* mutant deficient in *wxcB* the O-antigen was missing completely (Steffens *et al*. 2016). Another unusual gene of region 2 was *wxcD*, where a mutant strain (Vorhölter, Niehaus and Pühler 2001) produced LPS with fragmentary O-antigen (Braun *et al*. 2005), relating this gene possibly to O-antigen primer or adapter biosynthesis. Mutagenesis of the genes *wxcK* and *wxcN* (Steffens *et al*. 2016) in region 3 of the *wxc* gene cluster indicates a distinct biosynthetic mechanism for the synthesis of the O-antigen side chain.

### Cyclic glucans

Xanthomonads produce low-molecular-weight β-glucans including a cyclic *β*-glucan that is sequestered in the periplasmic space (Miller and Salama 2018). These molecules have been classified as membrane-derived oligosaccharides (MDOs). The cyclic *β*-glucan structures of *X*. *campestris* pv. *glycines* (*Xcg*) PDDCC 7414 and *Xac* have been confirmed as a *β*-(1→2)-linked glucan with 15 *β*-(1→2)-linkages and one *α*-(1→2)-linkage (York 1995). The cyclic *β*-(1→2)-glucan of *Xcc* has a role in the systemic suppression of plant immune responses (Rigano *et al*. 2007a) and may also protect bacteria against iron-induced toxicity (Javvadi *et al*. 2017).

### Regulation of cell surface carbohydrate biosynthesis

While the biosynthesis of cyclic glucan in *Xcc* appears to be regulated by the DSF system (Vojnov *et al*. 2001b), both DSF and DF signalling systems have an influence on xanthan biosynthesis. As outlined previously, the DSF system influences the levels of the second messenger cyclic di-GMP via several *rpf* gene products (O'Connell *et al*. 2013). The CAP-like protein (CLP), binds directly to the promoter region upstream of *gumB* in a manner modulated by the nucleotide second messenger (Chen *et al*. 2010a). It is not known how DSF regulates cyclic glucan synthesis or how DF signalling influences xanthan. There are additional regulatory effectors of *gum* expression: the GntR family regulator HpaR1 is an activator of *gum* gene expression that interacts directly with the *gumB* promoter (Su *et al*. 2016a) and CysB was recently identified as another transcriptional regulator that is likely to modulate *gum* gene expression (Schulte *et al*. 2019). These effects of DNA-binding transcriptional regulators are likely complemented by antisense transcripts that have been detected with several start sites, most outstanding for the promoter region upstream of *gumB* (Alkhateeb *et al*. 2016). In addition, proteomics data revealed that besides transcriptional regulation the post-translational modification of key enzymes may influence xanthan biosynthesis, in particular at the level of NDP-sugar synthesis from central metabolic intermediates (Musa *et al*. 2013). More research will be required to better understand this complex of regulatory influences.

## XANTHOMONAS DISEASE MANAGEMENT AND CONTROL

A suite of strategies for prevention and suppression of *Xanthomonas*-caused diseases have been developed; these have involved inhibition of pathogen dispersal, use of chemical and biocontrol agents and development and deployment of resistant varieties.

### Application of bactericides and biocontrol agents

For more than a century, copper and copper-based antimicrobial compounds were the main chemical control agents of *Xanthomonas* caused diseases. Currently, copper applications are widely used in the field and the compound is still considered as the most efficient means to control bacterial diseases (Lamichhane *et al*. 2018). However, in the past few decades many concerns have arisen regarding the sustainability and environmental effects of copper leading to the search for other control methods (Lamichhane *et al*. 2018). Resistance to copper has become more and more prevalent within *Xanthomonas* field isolates from different crops and locations. This resistance is attributed to the acquisition of copper resistance gene cluster through HGT (Bender *et al*. 1990; Cooksey 1994; Behlau *et al*. 2008). Copper resistance is usually plasmid encoded, and the plasmid is thought to be readily conjugated into other bacteria (Basim *et al*. 1999). Different strategies have been deployed to provide an efficient and sustainable substitute for copper and several products are now commercially available as biocontrol agents for *Xanthomonas* caused diseases (Table 2).

**Table 2.** Examples of commercially available biocontrol agents used for Xanthomonas-induced diseases.

Biocontrol agent	Commercial name/s	Diseases that can be controlled by the product ^A^	Approved for use				Comments
			USA^B^	EU^C^^,^^F^	Brazil^D^	China^E^	
Acibenzolar-s-methyl (ASM)	ACTIGARD^TM^ (Syngenta)	Broad range: Bacterial spot of tomato and pepper (*X. euvesicatoria, X. perforans, X. gardneri, X. vesicatoria*), Citrus canker (*X. citri* pv. *citri*), black rot of crucifers (*X. campestris* pv. *campestris*), bacterial spot of cucurbits (*X. cucurbitae*)	**√**	**√**	**√**	unknown	
	BION^R^ (Syngenta)						
	CGA 245704^TM^ (Syngenta)						
**Bactericides**							
Bismerthiazol		Bacterial blight of rice (*X. oryzae* pv. *oryzae*), bacterial streak of rice (*X. oryzae* pv. *oryzicola*)				**√**	Reported as a thyroid toxicant (Zhang, Wang and Zhu 2009), not approved use in multiple countries due to possible toxicity
Streptomycin	FireWall ^TM^ (Agrosource)	Bacterial spot of tomato and pepper (*X. euvesicatoria, X. perforans, X. gardneri, X. vesicatoria*), citrus canker (*X. citri* pv. *citri*)	**√**			**√**	High resistance reported in tomato infecting *Xanthomonas* strains (Sundin and Wang 2018)
Oxytetracycline	FireLine^TM^ (Agrosource)	Bacterial spot of peaches and nectarines (*X. arboricola* pv. *pruni*)	**√**				
	Mycoshield^TM^ (Nufarm Americas)						
Kasugamycin	Kasumin 2 L, (Arysta)	In USA: walnut blight (*X. arboricola* pv. *juglandis*) In China: black rot of crucifers (*X. campestris* pv. *campestris*), citrus canker (*X. citri* pv. *citri*), bacterial spot of peaches and nectarines (*X. arboricola* pv. *pruni*)	**√**			**√**	
**Bacteriophages**							
	AgriPhage^TM^ tomato spot & speck (omnilytics)	Bacterial spot of tomato and pepper (*X. euvesicatoria, X. perforans, X. gardneri, X. vesicatoria*)	**√**				
	AgriPhage™ citrus canker (omnilytics)	Citrus canker (*X. citri* pv. *citri*)	**√**				
**Bacterial control agents**							
*Bacillus pumilus* QST 2808	Sonata (Wilbur-Ellisa^®^)	Broad range	**√**	**√**	**√**		
*Bacillus amyloliquefaciens* QST 713	SerenadeASO (AgraQuest^®^)		**√**	**√**	**√**		
*Bacillus amyloliquefaciens* D747	CX 9030 (Agrobase)		**√**	**√**	**√**		
*Bacillus amyloliquefaciens* Strain FZB24	TAEGRO^®^ (Syngenta)	Bacterial spot of tomato and pepper (*X. euvesicatoria, X. perforans, X. gardneri, X. vesicatoria*)	**√**	**√**			
*Bacillus amyloliquefaciens* MBI 600	Serifel® Biofungicide (BASF)	Black rot of crucifers (*X. campestris* pv. *campestris*)		**√**			
*Bacillus amyloliquefaciens* B7900		Broad range				**√**	
*Bacillus amyloliquefaciens* PQ21						**√**	
*Bacillus amyloliquefaciens* B1619						**√**	
*Bacillus amyloliquefaciens* LX-11		Bacterial leaf streak of rice (*X. oryzae* pv. *oryzicola*), broad range				**√**	
*Streptomyces lydicus* WYEC 108	Actinivate (Novoenzymes)	Walnut blight (*X. arboricola* pv. *juglandis*), bacterial spot of tomato (*X. perforans*), citrus canker (*X. citri* pv. *citri*), angular leaf spot of strawberries (*X. fragariae*)	**√**	**√**			

A According to company specification sheet.

B Approved by United States Environmental Protection Agency, US EPA.

C Approved by EU according to EU commission—EU Pesticides database.

D Approved by the Brazilian Health Regulatory Agency (Anvisa).

E Approved by ministry of Agriculture of the People's Republic of China. We thank Drs. Xiaoyong Yuan, Zhigang Ouyang, Mingqing Shang, and Shengqi Chi for their help in collecting information.

F Some products approved by EU commission but is not yet authorised for use on a national level for each country.

Several types of antimicrobials were approved to be used for control against certain bacterial diseases (Table 2). Regulations of which compound can be used vary between countries and introduction of new antimicrobial agents in the field is subject to extensive testing prior to approval. Different antibiotics have been used against *Xanthomonas* caused diseases with some success. Streptomycin was first introduced as an agricultural antimicrobial agent in the 1950’s for treatment of bacterial diseases in various crops. The application of streptomycin, however, is soon followed by high frequency of resistance to the compound in different *Xanthomonas* spp. (Sundin and Wang 2018). Oxytetracycline is currently used for treatment of bacterial spot of peach caused by *X. arboricola* pv*. pruni*. This antibiotic treatment is effective for disease control in peach and no resistance to the treatment has been reported. The quinolone antibiotic oxolinic acid was reported to be successful in initial field trials against *X. frageria* and other bacterial pathogens such as *Erwinia amylovora* and *Burkholderia glumae* (Kim *et al*. 2016). However, this treatment failed to sustain its effect in long-term application because of the occurrence of resistant *E. amylovora* and *B. glumae* strains (Stockwell and Duffy 2012). Kasugamaycin had demonstrated some positive effect on *Xanthomonas* bacterial spot of pepper and tomato (Vallad *et al*. 2010) and was recently approved as a pesticide in the USA (Table 2). Field trials have been conducted to examine the effectiveness of the compound against other *Xanthomonas* caused diseases.

The potential application of antimicrobial peptides (AMPs) as a biocontrol agent for *Xanthomonas* treatments is currently under investigation. AMPs naturally occur in all forms of life and play a role in defence or competition against or between microorganisms (Hancock and Diamond 2000). AMPs biochemical properties give them semi-differential but usually not very specific toxic activity against multitude of microorganisms. Several AMPs are currently subjected to clinical trials for potential medicinal uses (Mahlapuu *et al*. 2016). Multiple studies have tested the potential application of natural and synthetic AMPs against bacterial and fungal pathogens, including *Xanthomonas* spp. and showed promising preliminary data. Studies have demonstrated high toxicity of AMPs on various *Xanthomonas* spp. *in vitro* while showing minimal effect on plant or mammalian cells (Choi and Moon 2009; Zeitler *et al*. 2013; Inui Kishi *et al*. 2018; Camó *et al*. 2019). Peptides were also tested for inhibition of plant diseases in greenhouse conditions in a few studies (Choi and Moon 2009; Shi *et al*. 2016).

Metal ions such as Zn^2+^ or Ag^+^ were previously reported to have an antimicrobial effect. However, the amount required for antimicrobial activity is high and will potentially have a negative environmental effect. Recent advances in nanoparticle technology should enable effective modification resulting in a more potent product. Accordingly, different metal-ion-based nanoparticles have been successfully utilised as antimicrobial compound in greenhouse and field trials against tomato infecting *Xanthomonas* strains (Ocsoy *et al*. 2013; Paret *et al*. 2013; Makarovsky *et al*. 2018; Liao *et al*. 2019).

### Bacteriophages

Bacteriophages are bacterial viruses that occur in almost all bacteria and usually have high host specificity. Soon after their discovery in the beginning of the 20th century the potential use of these viruses as antimicrobial agents was suggested; greenhouse-based studies displayed effective prevention of plant bacterial diseases as early as the mid-1920s (Abedon *et al*. 2011). An interest in utilising bacteriophages for biocontrol as an alternative to copper has arisen again in the past two decades. Many studies have been dedicated to the identification of bacteriophages in different *Xanthomonas* spp. and the utilisation of them against the bacterial pathogens in the field (Buttimer *et al*. 2017). Bacteriophages were indeed found to be effective in suppressing *Xanthomonas* caused diseases in tomato, citrus and onion in field conditions (Obradovic *et al*. 2004; Lang, Gent and Schwartz 2007; Balogh *et al*. 2008). While promising, there are limiting factors to bacteriophage application in the field to include their stability in the different environments and sensitivity to UV radiation.

### Systemic acquired resistance inducers

Several chemicals identified to actively induce systemic acquired resistance (SAR) have been utilised for management of diseases through activation of plant defence responses. The application of these inducers has a broad effect against many types of pathogens and in particular can effectively inhibit *Xanthomonas* diseases in multiple crops in the field (Thakur and Sohal 2013; Bektas and Eulgem 2015). A range of chemicals can induce SAR, although the exact mechanism of induction is not completely understood in most cases (Thakur and Sohal 2013; Bektas and Eulgem 2015). The main chemical compounds identified as SAR inducers are abicenzolar-S-mehtyl (ASM), Probenazole, *β*-amino butyric acid (BABA) and bismerthiazol. ASM has been commercialized (Table 2) and has been shown to be effective against many *Xanthomonas*-induced diseases (Obradovic *et al*. 2004; Gent and Schwartz 2005; Francis *et al*. 2009).

### Biocontrol through beneficial microorganisms

Naturally occurring bacteria from the plant rhizosphere and phyllosphere have been shown to affect plant pathogens and disease development (Parnell *et al*. 2016). A lot of work has been dedicated towards identifying isolates with antimicrobial-producing or SAR-inducing activity and utilising these bacteria to combat *Xanthomonas*-induced diseases. Plant-associated microorganisms exhibiting antimicrobial activity are attractive candidates for field biocontrol agents. *Bacillus* and *Streptomyces* spp. in particular, are considered as good candidates because of established antimicrobial attributes and sporulation capabilities. Indeed, many *Bacillus* and *Streptomyces* strains have been shown to have a broad range biocontrol effect (Shafi, Tian and Ji 2017; Vurukonda, Giovanardi and Stefani 2018) and several have been commercialised (Table 2). Numerous studies have demonstrated that foliar treatment with commercial or naturally isolated bacilli and *Streptomyces* strains significantly reduced *Xanthomonas* caused diseases in multiple crops in field trials (Van Hop *et al*. 2014; Thapa and Babadoost 2016; Daranas *et al.*^2019^).

Many bacterial isolates were reported to exert biocontrol through SAR induction. Repeated applications of *P. fluorescens* by foliar spray suppressed the disease severity of bacterial blight in repeated field trials and improved yield (Vidhyasekaran *et al*. 2001). Several rhizosphere isolates of *Burkholderia* and *Stenotrophomonas geniculata* with SAR-inducing traits successfully inhibited *X. citri* infection of grapefruit through soil drench treatment in greenhouse experiments (Riera *et al*. 2018).

Biocontrol of pathogenic *Xanthomonas* through non-pathogenic or attenuated *Xanthomonas* strains has also been examined. The general hypothesis behind using these non-pathogenic or attenuated strains is that pathogenic and non-pathogenic strains occupy same niches, but non-pathogenic strains induce SAR or utilise species specific bacteriocins against the virulent strains. Field trials utilising mutants in the type III secretion system that potentially elicit SAR indeed displayed strong biocontrol inhibition of *X. euvesicatoria* and *X. oryzae* on tomato and rice, respectively (Dardick *et al*. 2003; Moss *et al*. 2007). Non-pathogenic isolates of *X. campestris* that occur naturally have been shown to exert biocontrol activity against *X. arabicola* pv. *pruni* (*Xap*) in peach under greenhouse conditions (Kawaguchi, Inoue and Inoue 2014). While displaying high biocontrol capability during co-inoculation, the population of the non-pathogenic strains was not sustained in the host for long periods of time, questioning the sustainability of these strains in the field (Kawaguchi, Inoue and Inoue 2014). To overcome the inability to sustain an *in planta* population of non-pathogenic strains and type III secretion system mutants, Hert *et al*. attempted to use a bacteriocin-producing attenuated *X. perforans* strain to control bacteriocin sensitive *X. euvesicatoria* strains in tomato. The assumption here was that the attenuated strain would sustain a stable population that would be insufficient to induce disease symptoms while nevertheless inhibiting the pathogenic strain (Hert *et al*. 2009). Although the pathogenic strain was inhibited, it was not completely eradicated, however, and multiple applications of the biocontrol strain were required to sustain inhibition (Hert *et al*. 2009).

### Generation of resistant varieties

Generation of disease resistant varieties is one of the most efficient, sustainable, environmentally friendly and desired disease control approaches. Plant resistance or susceptibility to certain pathogens have been demonstrated to be a hereditable trait. Many cultivar specific *Xanthomonas* resistance loci were identified in different plants. The number of resistance genes identified in domesticated varieties varies greatly between plant species. For instance, dozens of *Xanthomonas* resistance associated loci have been identified in rice (Khan, Naeem and Iqbal 2014) while to date none have been identified in other crops, such as banana, peach or citrus,.

Resistance to *Xanthomonas* spp. can be multiple quantitative trait loci (QTL) or can originate from a single dominant or recessive locus. In most cases, a dominant locus represents active recognition of pathogen determinant by a resistance (R) or pattern recognition receptor (PRR) proteins while recessive loci are usually associated with the modulation of pathogen susceptibility genes. One of the well characterised recessive resistance loci against *Xanthomonas* was found to induce the expression of the *SWEET* transporter family genes, which are named susceptibility (S) genes, in rice by *X. oryzea* TAL effectors (Hutin *et al*. 2015a; Ji, Wang and Zhao 2018). The effector-binding element (EBE) of *SWEET* genes is mutated in rice R loci *xa13*, *xa25* and *xa41* (Chu *et al*. 2006; Yang, Sugio and White 2006; Liu *et al*. 2011; Hutin *et al*. 2015b). In addition, reduced affinity of TAL effectors to transcriptional machinery was identified as the functional mechanism of *xa5* resistance locus (Yuan *et al*. 2016).

A handful of dominant resistance genes against *Xanthomonas* spp. were cloned from pepper, rice, tomato and maize and the bacterial determinants were identified in some of them. The majority of such genes were identified to be intracellular NB-LRR receptor genes (pepper *Bs2*, maize *Rxo1*, rice *Xa1* and tomato *Bs4*) or outer-membrane receptor genes (rice *Xa21*, *Xa3*/*Xa26* and *Xa4*) (Table S3, Supporting Information). Dominant executor *R* genes (pepper *Bs3* and *Bs4C* and rice *Xa10*, *Xa23*, and Xa27) target specific TAL effectors. Executor genes encode hypersensitive-response inducing proteins under a promoter region that contains the EBE of virulent TALs (Zhang, Yin and White 2015) (Table S3, Supporting Information). Even though considerable effort has been dedicated to the identification and characterisation of hereditable resistance traits, the majority of genes conferring resistance are unknown. In many cases, a resistance trait is identified and even mapped to a certain chromosomal region but the resistance gene itself is not identified (Khan, Naeem and Iqbal 2014) (Table S4, Supporting Information). Many virulence determinants, mainly T3Es, have been shown to dictate cultivar and even species host specificity but the plant *R* genes responsible for the resistance have not been cloned (Table S4). Identification, cloning and characterisation of these unknown *R* genes can greatly contribute to the development of new *Xanthomonas* resistant varieties that will increase yield in the field.

### Generation of resistant varieties through traditional breeding

Resistance to pathogens is a major trait to be considered during breeding. Substantial efforts are being made to identify and introduce resistant or tolerant accessions for breeding against disease. However, in many cases, the screened resistant lines are either significantly different from the main commercial lines or are wild germplasms of a domesticated crop. The introduction of resistant genes into desired domesticated crops requires significant amount of crossings to produce near isogenic lines, a task that is not completely realistic in perennial tree plants or hybrid lines that are vegetatively propagated such as most domesticated citrus varieties (Wu *et al*. 2018). Nevertheless, genes conferring resistance against *Xanthomonas* have been introduced through traditional breeding to produce near-isogenic lines of rice, pepper and tomatoes (Stall, Jones and Minsavage 2009) (Chukwu *et al*. 2019). Cross-breeding procedures are currently ongoing for many annual crops such as strawberries, maize, cassava and wheat (Roach *et al*. 2016; Soto Sedano *et al*. 2017; Wen *et al*. 2018).

Durability is also a significant factor in the introduction of resistant varieties. Many *Xanthomonas* races within a species are found to infect resistant lines and in many cases quick field adaptation of the pathogen occurs (Stall, Jones and Minsavage 2009; Hutin *et al*. 2015a). For example, resistance to TAL effectors that is achieved through alterations in the promoters of *S* gene or utilisation of executor *R* genes are usually specific to certain pathogen races and can be easily overcome by *Xanthomonas* pathogens (Hutin *et al*. 2015a). *X*anthomonas *oryzae* races contain multiple TALs that target different promoter regions in the same S gene or alternatively target a different *S* gene with similar function (Hutin *et al*. 2015a).

The majority of non-TAL effectors are functionally redundant and a single effector is usually not significant for pathogenicity (Hajri *et al*. 2009; Cunnac *et al*. 2011). For pepper or tomato, *Xanthomonas* bacteria were able to quickly overcome the introduction of non-TAL recognising resistant genes (pepper *Bs1* and tomato *Xv3* and *rx1*, *rx2* and *rx3*) in the field through the loss or inactivation of the avirulence effector and shift in pathogen races (Stall, Jones and Minsavage 2009). On the other hand, the non-TAL effector gene *avrBs2* is highly conserved in *Xanthomonas* spp. (Hajri *et al*. 2009) and is required for the virulence of many *Xanthomonas* strains such *X. euvesicatoria*, *Xac* and *Xam* (Li *et al*. 2015; Medina *et al*. 2018). Consequently, the pepper *Bs2* locus, which confer resistance upon recognition of AvrBs2, serves as a good candidate for durable field resistance. Production of near isogenic lines of pepper containing *Bs2* was indeed found to give relatively stable field resistance to bacterial spot, however, few *X. euvesciatoria* isolates were identified in the resistant lines and mutation in *avrBs2* in relatively low rate frequency was identified in *Xanthomonas* mutant collections (Wichmann *et al*. 2005).

Pyramiding disease resistance genes against *Xanthomonas* has been conducted in pepper, cotton and rice (Delannoy *et al*. 2005; Stall, Jones and Minsavage 2009; Chukwu *et al*. 2019). Pepper near isogenic lines ECW123 (containing *Bs1*, *Bs2* and *Bs3*) were generated and indeed showed enhanced resistance to bacterial spot but still could not provide complete protection against the *Xanthomonas* pathogen (Stall, Jones and Minsavage 2009). Several commercially available rice lines harbouring a combination of multiple resistance genes against bacterial blight caused by *Xoo* have been produced and introduced into the field (Chukwu *et al*. 2019). Improved Samba Mahsuri is a near isogenic line containing three bacterial blight resistance genes (*Xa21*, *xa13*, and *xa5*) in 2008 (Sundaram *et al*. 2009); thus far this line has provided high resistance against bacterial blight in India.

### Introduction of resistant varieties through genetic engineering

The introduction of genetically modified (GM) crops has long been suggested as an approach to overcome the obstacles of traditional breeding, in particular to enable modification of crops with long generation times that cannot be bred into near isogenic lines in a realistic time window. Producing GM crops does raise many issues. Regulation of production, consumption and research of GM crops and addressing public concerns regarding the potential health hazards or the economic effect of patenting agricultural products are among the limiting factors that should be considered before proceeding in that direction (Raman 2017; Thomas and De Tavernier 2017; Faure and Napier 2018). With that in mind, multiple approaches are being used for introduction of resistance or tolerance to *Xanthomonas* in commercial crops.

The most direct means to generate resistant transgenic plants is the introduction of *R* genes or immune receptors. General induction of immune response was proven efficient in laboratory conditions but is usually associated with other pleiotropic effects on plant physiology that might reduce yield in field conditions. The introduction of immune receptors might overcome such issues, but, similarly to the resistant lines in breeding, it can be associated with relatively fast adaptation of the pathogens. Several *R* or *PRR* genes from different plant species have been introduced into commercial crops. For example, introduction of the pepper *Bs2* or Arabidopsis *ELONGATION FACTOR TU RECEPTOR* (*EFR*) into tomato increases resistance to *Xanthomonas* bacterial spot in greenhouse and field trials (Horvath *et al*. 2012; Kunwar *et al*. 2018). Introduction of other resistance genes like pepper *Bs2*, rice *Xa21* and *N. benhtamiana FLAGELLIN SENSITIVE2* (*FLS2*) into citrus, maize *Rxo1* into rice and rice *Xa21* into banana inhibited *Xanthomonas* induced diseases in greenhouse and glasshouse conditions (Zhou *et al*. 2010; Tripathi *et al*. 2014; Hao *et al*. 2015; Sendín *et al*. 2017; Omar *et al*. 2018). Durability of these transgenic plants in the field is yet to be determined.

Another strategy for transgene-induced resistance is through promoter engineering of executor *R* genes. Zeng *et al*. engineered the promoter region of *Xa10* executor *R* gene to contain EBE recognition site of multiple *X. oryzae* TALEs and demonstrated that the modified executor *R* gene, *Xa10* (E5), provided broad spectrum resistance against 27 *X. oryzae* field isolated (Zeng *et al*. 2015). Using a similar approach, Shantharaj and colleagues introduced the EBE region of the *CsLOB1*, the canker susceptibility gene induced by *X. citri* PthA4, into the promoter of the *Bs3* gene and replaced *Bs3* with *avrGf1* which is recognised as an avirulence gene in grapefruit (Shantharaj *et al*. 2017). Transient expression of the engineered executor *R* gene in grapefruit leaves leads to the development of HR in response to *X. citri* and reduced bacterial colonisation (Shantharaj *et al*. 2017).

Few studies have introduced bacterial genes into crops to combat *Xanthomonas* diseases in greenhouse conditions. For example, Caserta and colleagues have introduced DSF producing gene *rpfF* from *Xylella* into citrus and demonstrated enhanced resistance to *X. citri*, possibly through misregulation of virulence-associated genes in an improper bacterial quorum (Caserta *et al*. 2014). In addition, Zhang and colleagues expressed *Pantoea albD*, which was shown to detoxify the *X. albilineans* toxin albicidin in sugarcane. This lead to a reduction of *X. albilineans* leaf scald symptoms with partial success (Zhang, Xu and Birch 1999).

*Xanthomonas*-insensitive rice and citrus were produced through targeting the EBE of *S* locus promoter. Li and colleagues utilized TALEN-based gene editing to modify the EBE region, recognized by two *X. oryzae* TAL effectors, of the rice *OsSWEET14* susceptibility gene (Li *et al*. 2012). Selfing of modified lines resulted in a non-transgenic rice plants with resistance to the *X. oryzae* strains (Li *et al*. 2012). Two groups independently disrupted the *X. citri* PthA4 EBE binding region of the citrus canker susceptibility gene *CsLOB1* using CRISPR/Cas9 system in grapefruit and Wangjincheng orange (Jia *et al*. 2016; Peng *et al*. 2017). The PthA4 EBE was disrupted in differential ratios in transgenic plants expressing the CRISPR/Cas9 system and, as expected, the plants displayed reduced canker symptoms when inoculated with *X. citri* (Jia *et al*. 2016; Peng *et al*. 2017). A novel technique developed by Murata and colleagues was based on production of cybrid cells containing combinations of nucleus and organelles from different cultivars (Murata *et al*. 2018). Using somatic hybridisation, the group produced a cybrid grapefruit containing the nucleus of *X. citri* susceptible grapefruit (*Citrus paradisi*) and the chloroplast of *X. citri* tolerant/resistant Meiwa kumquat (*Citrus japonica*). The cybrids displayed enhanced resistance to *X. citri* infection, thus demonstrating that organelle genetic background plays an important role in pathogen response (Murata *et al*. 2018).

## CONCLUDING REMARKS

Since the report of the first two *Xanthomonas* genomes almost 20 years ago, there has been an extensive body of work describing factors that contribute to *Xanthomonas* disease in their respective host plants. It is now clear that *Xanthomonas* has developed a range of highly regulated and co-ordinated traits needed to adhere to plant tissue, acquire nutrients, suppress plant defence responses and ultimately cause disease. In spite of these detailed studies of molecular mechanisms underlying the activity of *Xanthomonas* virulence factors there are still many unanswered questions. Some of the major challenges include the understanding *Xanthomonas* tissue specificity and preference for portal of entry mechanisms of sensing of plant-derived signals; the elucidation of virulence-associated regulatory systems, which include RNA-binding proteins and small RNAs. It is believed that multidisciplinary approaches (comparative genomics, functional genomics, population genomics, metagenomics, transcriptomics, proteomics and systems biology) will be deployed in the future research to address these issues.

Efforts are dedicated to understanding *Xanthomonas* ecology, epidemiology, and phytopathology have the ultimate goal of controlling or suppressing plant disease. This knowledge has begun to be translated into various strategies including pathogen chemical inhibition of dispersal, biocontrol agents and utilisation of resistant varieties. In addition to these approaches, the growing appreciation of the role of the plant microbiome in promoting plant health and growth suggests the possibility of manipulation of endophytic/epiphytic communities to achieve some measure of disease control. However, studies of the impact of the microbiome on *Xanthomonas* colonisation and disease control are currently at an early stage.

## Supplementary Material

fuz024_Supplemental_File
