# Supplementary material for: Mechanistic insights into host adaptation, virulence and epidemiology of the phytopathogen Xanthomonas
Source: FEMS Microbiol Rev. 2019 Oct 3;44(1):1–32. doi: 10.1093/femsre/fuz024 (PMC8042644; doi:10.1093/femsre/fuz024)
Supplement: fuz024_Supplemental_File [file fuz024_supplemental_file.docx]

**Mechanistic insights into host adaptation, virulence and epidemiology of the phytopathogen *Xanthomonas***

Shi-qi An, Neha Potnis, Max Dow, Frank-Jörg Vorhölter, Yong-qiang He, Anke Becker, Doron Teper, Yi Li, Nian Wang, Leonidas Bleris, Ji-liang Tang

Correspondence should be addressed: jitang@gxu.edu.cn

**Table S1.** Known type III effectors of found in Xanthomonas species and their (predicted) function. This list only includes effectors with a demonstrated role in pathogenicity or induction of resistance.

**Table S2.** Summary of proteins that involved in signaling and regulation of virulence in *Xanthomonas* species

**Table S3.** List of cloned dominant resistance genes effective against *Xanthomonas* spp.

**Table S4.** *Xanthomonas* effector genes with unknown corresponding R genes

**Table S1.** Known type III effectors found in *Xanthomonas* species and their (predicted) function. This list only includes effectors with a demonstrated role in pathogenicity or induction of resistance.

| **Effector class** | **Family** | **Synonyms** | **Distribution** | **PIP box** | **Features** | **Function on pathogenicity** |
| --- | --- | --- | --- | --- | --- | --- |
| **Core effectors*** |  |  |  |  |  |  |
| XopR | XopR |  | All *Xanthomonas* spp. except *Xal* | Yes | Unknown | Suppresses PAMP-Triggered Immunity |
| AvrBs2 | AvrBs2 |  | All *Xanthomonas* spp. except *Xca* and *Xal* | Yes | Sequence related to agrocinopine synthase, glycerol phosphodiesterase | Avirulence in *Brassica juncea*, *B. carinata* and *B. oleracea* and HR on pepper or no effect, suppresses rice immunity |
| XopK | XopK |  | All *Xanthomonas* spp. except *Xca* and *Xal* | Yes | Unknown | Unknown |
| XopL | XopL |  | All *Xanthomonas* spp. except *Xca* and *Xal*  non-canonical version in *Xcc* 8004,33913  (shorten version) | Yes | LRR protein, E3 ubiquitin ligase | Targets the role of microtubules in stromule extension and dynamics |
| XopN | XopN |  | All *Xanthomonas* spp. except *Xca* and *Xal* | Yes | ARM/HEAT repeat | Suppresses PAMP-Triggered Immunity |
| XopP | XopP |  | All *Xanthomonas* spp. except *Xal* | Yes | Unknown | Modulates host E3 ligase activity and suppresses PAMP-triggered immunity in rice |
| XopQ | XopQ |  | All *Xanthomonas* spp. except *Xca* and *Xal* | Yes | Putative inosine-uridine nucleoside N-ribohydrolase | Suppresses effector-triggered immunity |
| XopX | XopX |  | All *Xanthomonas* spp. except *Xca* and *Xal* | Yes | Methionine-rich protein | Suppresses and activates plant defense responses |
| XopZ | XopZ1 |  | All *Xanthomonas* spp. except *Xca* and *Xal* | Yes | Unknown | Suppresses innate immune responses |
| **Variable effectors*** |  |  |  |  |  |  |
| XopJ | XopJ2 | AvrBsT | Some *Xav* strains (plasmid borne) | Yes | C55 family cysteine protease, Ser/Thr acetyl transferase |  |
| XopC | XopC1 |  | Only in *Xav* (*xopC*);  Only in *Xoo* strains (*xopC*2-inactivated version in *Xcci*, *Xav*) | Yes | Phosphoribosyl transferase domain, haloacid dehalogenase-like hydrolase |  |
| XopF | XopF1 | Hpa4 | *Xav, Xca, Xoo, Xoc, Xvv, Xvm*. | Yes | Unknown |  |
| XopF | XopF2 |  | *Xav, Xvm, Xvv* | - | Unknown |  |
| XopJ | XopJ1 |  | *Xav* | Yes | C55-family cysteine protease or Ser/Thr acetyltransferase |  |
| XopO | XopO |  | *Xav, Xoc* | Yes | Unknown |  |
| XopAE | XopAE | HpaF/HpaG | *Xag, Xcci, Xoo, Xoc, Xvm, Xvv*. (*hpaG/hpaF* pseudogene in *Xav*) | Yes | Leucine-rich repeats |  |
| XopAH | XopAH | AvrXccC | Only in *Xcc* | Yes | Unknown | Avirulence on *B. rapa* |
| XopE | XopE2 | AvrXacE3, AvrXccE1 | *Xav, Xcci* and *Xcc*  Diversifying in N-terminus | Yes | Putative transglutaminase;  Depending on the conserved myristoylation motif ;  XopE2 is phosphorylated at multiple residues in planta  Intereacting wih 14-3-3 isoform (TFT) protein | Suppress symptom in susceptible hosts (putative avirulence on *B. rapa*) |
| XopAM | XopAM |  | *Xcc, Xvm, Xvv*. | Yes | Unknown | Avirulence on *Arabidopsis thaliana* |
| AvrBs3 | AvrBs3 | Pth, TAL | *Xcci, Xoo, Xoc, Xcc, Xav, Xg* | - | Transcriptional activator like | Avirulence or virulence on rice (*Oryzae sativa*), causes canker-like symptoms in citrus, increased release of the pathogen to cotton leaf surfaces |
| XopD | XopD |  | *Xav, Xcc* str. B100. Chimeric version in *Xcc* str. 33913, 8004 | Yes | C48-family small ubiquitin-like modifier (SUMO) cysteine protease; EAR motif; DNA-binding and nuclear localization domain | Triggers disease tolerance and increase bacterial survival in Arabidopsis |
| AvrBs1 | AvrBs1 |  | *Xcc, Xav* | - | Unknow | Avirulence on *Nicotiana* *benthamiana.*Recognized by Bs1 and induced HR on pepper ECW-10R. |
| XopB | XopB |  | *Xav, Xvm* | Yes | Unknown | Suppressing defense responses related to both PAMP-triggered immunity (PTI) and effector-triggered immunity, interferes with eukaryotic vesicle trafficking. |
| XopH | XopH1 | AvrBs1.1 | *Xcc, Xav* | - | Putative tyrosine phosphatase | Suppresses PAMP-triggered immunity, inhibited flg22-induced callose deposition. |
| XopS | XopS |  | *Xcci, Xav* | - | Unknow | Suppresses PAMP-triggered immunity. |
| XopY | XopY |  | *Xoo, Xcc, Xvm* | Yes | Unknow | Targeted OsRLCK185, inhibited peptidoglycan- and chitin-induced immunity and pathogen resistance. |
| XopAA | XopAA |  | *Xoo, Xcv, Xoc* | Yes |  | May interacted with OsBAK1 and suppress host immunity in rice. Triggered host immune response in *Arabidopsis thaliana*. |
| XopAJ | XopAJ | AvrRxo1 | *Xav, Xoc* | Yes |  | Suppresses PAMP-triggered immunity, inhibited flg22-induced callose deposition. Recognised by non-host resistance protein Rxo1, triggered (HR) in maize. |
| XopAC | XopAC | AvrAC | *Xcc, Xcr* | Yes | LRR-Fic/DOC protein | In susceptible Arabidopsis plants, XopAC type III effector inhibits by uridylylation positive regulators of the PAMP-triggered immunity such as the receptor-like cytoplasmic kinases (RLCK) BIK1 and PBL1. In the resistant ecotype Col-0, xopAC is a major avirulence gene of Xcc. |
| XopAU | XopAU |  | *Xcci, Xav, Xoo, Xoc* | Yes | Serine/threonine kinase | Manipulated plant MAP kinase signalling by directly phosphorylated MKK2. |

**Note:** Core effectors are found in almost all *Xanthomonas* spp. with the exception of *Xal* (which has none) and *Xca* (which has only XopR and XopP). Variable effectors are found in only a limited number of strains.

**Table S2.** Summary of proteins that involved in signalling and regulation of virulence in *Xanthomonas* species

|  | ***Xanthomonas campestris* pv. *campestris* 8004** | ***Xanthomonas citri* pv. *citri 306*** | ***Xanthomonas oryzae* pv. *oryzae***  **PXO99A** | ***Xanthomonas oryzae* pv. *oryzicola* BLS256** | **Brief functional description** |
| --- | --- | --- | --- | --- | --- |
| **Cell-cell signalling and associated pathways** | | | | | |
| RpfC-RpfG | XC_2333-XC_2335 | XAC1878-XAC1877 | PXO_00069 | XOC_2265-XOC_2264 | Involved in DSF cell-cell signalling, virulence, biofilm formation |
| RpfF | XC_2332 | XAC1879 | PXO_00070 | XOC_2266 | Bifunctional crotonase involved in the synthesis of DSF family signalling molecules |
| RpfB | XC_2331 | XAC1880 | PXO_00067 | XOC_2267 | Involved in the synthesis of DSF family signalling molecules |
| RpfS | XC_2579 | XAC1669 | Absent | XOC_3430 | Involved in DSF cell-cell signalling that controls virulence factors |
| XanB2 | XC_4103 | XAC4103 | PXO_03739 | XOC_0424 | A bifunctional chorismatase involved in DF signalling |
| **Intracellular signalling mediated by nucleotide second messengers** | | | | | |
| Clp | XC_0486 | XAC0483 | PXO_04006 | XOC_4202 | A c-di-GMP effector and transcriptional regulator involved in virulence and biofilm formation |
| YajQ | XC_3703 | XAC3671 | PXO_03091 | XOC_3939 | A c-di-GMP effector involved in virulence and biofilm formation |
| PilZ | XC_0965 | XAC3402 | PXO_02374 | XOC_3659 | A c-di-GMP effector involved in virulence and motility |
|  | XC_2249 | XAC1971 | PXO_00997 | XOC_2370 | A c-di-GMP effector involved in virulence and motility |
|  | XC_3221 | XAC1133 | PXO_02715 | XOC_1181 | A c-di-GMP effector involved in virulence and motility |
|  | XC_1824 | XAC2398 | PXO_00403 | XOC_2102 | A c-di-GMP effector involved in virulence and motility |
|  | XC_0249 | XAC0258 | PXO_00649 | XOC_2989 | A cGMP effector in virulence and biofilm formation |
|  | XC_0250 | XAC0259 | Absent | Absent | A cGMP synthase involved in virulence |
| FimX | XC_1824 | XAC2398 | PXO_00403 | XOC_2102 | A c-di-GMP signalling protein with DDEF and EAL domains involved in pilus development |
| **Two-component systems** | | | | | |
| RaxH-RaxR | XC_3125-XC_3126 | XAC1222-XAC1221 | PXO_04467- PXO_04469 | XOC_1287-XOC_1286 | Regulation of AvrXa21 activity and virulence |
| PhoQ-PhoP | XC_4030-XC_4031 | XAC4022-XAC4023 | PXO_02837- PXO_02836 | XOC_4318-XOC_4319 | Regulation of virulence |
| ColS-ColR(VgrS-VgrR) | XC_1050-XC_1049 | XAC3249-XAC3250 | PXO_02305-  PXO_02303 | XOC_3522-XOC_3523 | Modulates bacterial responses to iron-replete and depleted conditions |
| RavS-RavR | XC_2227-XC_2228 | XAC1994-XAC1992 | PXO_01020- PXO_01019 | XOC_2394-XOC_2393 | Involved in sensing low-oxygen tension and virulence regulation |
| RavA-RavR (PdeK-PdeR) | XC_2229-XC_2228 | XAC1991-XAC1992 | PXO_01018-  PXO_01019 | XOC_2392-XOC_2393 | Regulates virulence and exopolysaccharide production |
| HpaR2-HpaS | XC_3669-XC_3670 | Absent-XAC3643 | Absent | XOC_3908-XOC_3909 | Regulates virulence |
| HpaS-hprG | XC_3670-XC_3077 | XAC3643- XAC1265 | Absent -PXO_01951 | Absent | Regulates virulence |
| PcrK-PcrR | XC_1756-XC_1755 | XAC2492-XAC2493 | Absent | XOC_1985-XOC_1984 | Senses the plant hormone cytokinin to promote adaptation to oxidative stress |
| SreK-SreR-SreS | XC_0728-XC_0729-XC_0730 | XAC0683-XAC0684-XAC0685 | PXO_04304-PXO_04305-PXO_04306 | XOC_0743-XOC_0744-XOC_0745 | Coordinates virulence factor regulation |
| StoS | XC_3714 | XAC3683 | PXO_03078 | XOC_3954 | Coordinates virulence factor regulation |
| GsmR | XC_0850 | XAC3443 | PXO_02599 | XOC_3708 | Positively controlled by Clp and is involved in expression of genes responsible for flagellum synthesis |
| XerR (XmbR, XibR) | XC_3760 | XAC3733 | PXO_03020 | XOC_4004 | Regulates virulence associated and iron metabolism functions in response to iron availability |
| VemR | XC_2252 | XAC1968 | PXO_00994 | XOC_2367 | Positively regulates the virulence and adaptation |
| **TonB-dependent receptors (TBDRs)** | | | | | |
| NixB | XC_0543 | XAC0544 | PXO_02415 | XOC_3757 | Involved in uptake of GlcNAc-containing molecules derived from the plant |
| NixD | XC_1222 | XAC3071 | PXO_01644 | XOC_3256 | Involved in uptake of GlcNAc-containing molecules derived from the plant |
| NixC | XC_1165 | XAC3121 | PXO_01579 | XOC_3328 | Involved in uptake of GlcNAc-containing molecules derived from the plant |
| SuxA (fyuA) | XC_0806 | XAC3489 | PXO_02415 | XOC_3757 | Involved in sucrose uptake |
| NixA | XC_0756 | XAC0716 | PXO_01644 | XOC_3256 | Involved in uptake of GlcNAc-containing molecules derived from the plant |
| TBDR | XC_3706 | XAC4131 | Absent | XOC_3256 | Involved in hypersensitive response |
| **Light responsive sensors** | | | | | |
| BphP | XC_4241 | XAC4293 | PXO_03310 | XOC_0228 | Bacteriophytochrome photoreceptor that is involved in the regulation of virulence |
| **Sigma (σ) factors** | | | | | |
| RpoD | XC_3806 | XAC3788 | PXO_04069 | XOC_4120 | A factor involved in growth |
| RpoH | XC_3843 | XAC3824 | PXO_03711 | XOC_0605 | A factor involved in growth |
| RpoE1 | XC_2974 | XAC1319 | PXO_01711 | XOC_3188 | A factor involved in virulence regulation |
| RpoN1 | XC_1311 | XAC2972 | PXO_02227 | XOC_1439 | A factor involved in motility regulation |
| RpoN2 | XC_2251 | XAC1969 | PXO_00995 | XOC_2368 | A factor involved in motility regulation |

**Table S3.** List of cloned dominant resistance genes effective against *Xanthomonas* spp.

| **Resistance gene** | **Plant/variey** | **Accession (*R* gene)** | **Avirulance gene** | **Effective against**  ***Xanthomonas* strain/s** | **Accession (avirulance gene)** | **Comments** | **References** |
| --- | --- | --- | --- | --- | --- | --- | --- |
| Outer-membrane receptor family | | | | | | | |
| *Xa21* | Wild rice (*Oryza longistaminata*) | OSU37133 | RaxX | *X. oryzae* pv. *oryzae*, *X. campestris* pv. *musacearum*, *X. citri* pv. *citri* | AJQ82236 | Banana and citrus transgenic lines display resistance to *Xanthomonas* pathogens | (Song *et al.* 1995)(Pruitt *et al.* 2015) (Tripathi *et al.* 2014) (Mendes *et al.* 2010) |
| *Xa3/Xa26* | Rice (*Oryza sativa japonica*) | DQ426646 | Unknown | *X. oryzae* pv. *oryzae* |  |  | (Sun *et al.* 2004) |
| *Xa4* | Rice (*Oryza sativa Japonica*) | KU761313 | Unknown | *X. oryzae* pv. *oryzae* |  | Cell wall associated | (Hu *et al.* 2017) |
| NB-LRR family | | | | | | | |
| *Bs2* | Pepper (*Capsicum chacoense*) | AF202179 | AvrBs2 | *X. euvesicatoria, X. perforans, X. gardneri, X. citri* pv*. citri* | CAJ21683 | Tomato and citrus transgenic lines display resistance to *Xanthomonas* pathogens | (Kearney and Staskawicz 1990)(Tai *et al.* 1999)(Horvath *et al.* 2012)(Sendín *et al.* 2017) |
| *Bs4* | Tomato (*Solanum lycropersicum*) | AY438027 | TAL/Pth | *X. euvesicatoria, X. vesicatoria* | CAA48680 | Non-specific TAL effector recognition. Independent of RVD identity. | (Schornack *et al.* 2005)(Schornack *et al.* 2004) |
| *Rxo1* | Maize (*Zea mays*) | AY935244 | XopAH/AvrRxo1 | *X. oryzae* pv. *oryzicola* | AEQ98135 | From non-host plant. Rice transgenic lines display resistance to *X*. *oryzae* pv. *oryzicola* | (Zhao *et al.* 2004b)(Zhao *et al.* 2004a) |
| *Pbl2/Rks1/Zar1* | Arabidopsis thaliana (Col-0) | NM_101304,NM_114955, KF363734 | XopAC | *X. campestris* pv. *campestris* | AAY48619 | Function as a decoy complex | (Wang *et al.* 2015)  (Xu *et al.* 2008) |
| *Xa1* | Rice (*Oryza sativa Japonica*) | LOC4345202 | TAL/Pth | *X. oryzae* pv. *oryzae* | AAS75145 | Non-specific TAL effector recognition. Independent of RVD identity. | (Yoshimura *et al.* 1998)(Ji *et al.* 2016) |
| Executor family | | | | | | | |
| *Bs3* | Pepper  (*Capsicum annuum*) | EU078684 | AvrBs3, AvrHah1 | *X. euvesicatoria*, *X. gardneri, X. vesicatoria* | CAA34257, ABP97430 |  | (Römer *et al.* 2007)(Van den Ackerveken, Marois and Bonas 1996)(Schornack *et al.* 2008) |
| *Bs4C* | Pepper (*Capsicum pubescens*) | JX944826 | AvrBs4, AvrBsP | *X. euvesicatoria, X. vesicatoria* | CAA48680 |  | (Strauss *et al.* 2012)(Wang *et al.* 2018) |
| *Xa10* | Rice (*Oryza sativa Indica*) | JX025645 | AvrXa10 | *X. oryzae* pv. *oryzae* | Q56830 |  | (Tian *et al.* 2014) |
| *Xa23* | Rice (*Oryza sativa Indica*) | KP123635 | AvrXa23 | *X. oryzae* pv. *oryzae* | ADK73960 |  | (Wang *et al.* 2015) |
| *Xa27* | Rice (*Oryza sativa Indica*) | JN601064 | AvrXa27 | *X. oryzae* pv. *oryzae* | AAY54168 |  | (Gu *et al.* 2005) |

**Table S4.** *Xanthomonas* effector genes with unknown corresponding R genes

| **Effector** | **accession** | **strain** | **host** | **Comments** | **Reference** |
| --- | --- | --- | --- | --- | --- |
| XopAH/AvrXccC | AAY49067 | *X. campestris* pv. *campestris* | Mustard (*Brassica napiformis*) |  | (Wang, Tang and He 2007) |
| XopQ | CAJ26169, AGM16438 | *X. oryzae* pv. *oryzae*, *X. euvesicatoria*, *X. perforans* | *Nicotiana benthamiana* |  | (Gupta *et al.* 2014)(Schwartz *et al.* 2015) |
| XopJ2/AvrBsT | ANN45441 | *X. perforans* | *Nicotiana benthamiana* |  | (Schwartz *et al.* 2015) |
| XopJ2/AvrBsT | ANN45441 | *X. perforans* | *Arabidopsis thaliana* |  | (Cheong *et al.* 2014) |
| XopJ2/AvrBsT | ANN45441 | *X. perforans* | Pepper (*Capsicum annuum*) |  | (Kim, Choi and Hwang 2010) |
| AvrBs1 | CAJ19916, EGD20588 | *X. gardneri*, *X. euvesicatoria* (race 1) | Pepper (*Capsicum annuum* ECW-10R) | Hereditary dominant resistance locus (*Bs1*) was crossed into pepper commercial line ECW. *R* gene was not cloned. | (Ronald and Staskawicz 1988) |
| XopH/AvrBs7/avrBs1.1 | AUN35361, CAJ19917 | *X. gardneri, X. euvesicatoria* (race 1) | Pepper  (*Capsicum baccatum* var. *pendulum*) |  | (Potnis *et al.* 2011) |
| XopAF/AvrXv3 | ANN45471 | *X. perforans* (race T3) | Tomato (*Solanum lycopersicum* H7981) | Resistant locus area (*Xv3*) was mapped but *R* gene was not cloned. | (Wang *et al.* 2011) |
| XopJ4/AvrXv4 | WP_008572727 | *X. perforans* | Wild tomato (*Solanum pennellii*) | Resistant locus area (*Xv4*) was mapped but *R* gene was not cloned. | (Astua-Monge *et al.* 2000) |
| XopJ3/AvrRxv | CAJ22102 | *X. euvesictoria* | Tomato (*Solanum lycopersicum* H7998) | Resistant loci area (*Rx-1*, *Rx-2* and *Rx-3*) were mapped but never cloned. | (Whalen *et al.* 1993) |
| XopAG/AvrGf1/AvrGf2 | AIP90071, AGI06427 | *X. citri* pv. *citri* (AW strain), *X. fuscans* pv. *aurantifolii* (C strain) | Grapefruit (*Citrus paradisi*) |  | (Gochez *et al.* 2015) |
| Pths/TALs : PthN, AvrB101, AvrBIn, AvrB4, Avrb7, AvrB102, Avrb6, AvrB5 | ASN11708, ASN03458, ASN03465, ASN03443, ASN03489, ASN03473, AAB00675, ASM99420 | *X. campestris* pv. *malvacearum* | Cotton (*Gossypium* spp.) varieties: AcB2, AcB4, Acb6, Acb7, AcBln, Gregg, Ac2BS9, AcS295, AcB5b | Multiple TALs induced HR in different cotton varieties. Resistant mechanism is unclear. | (Feyter and Gabriel 1991) (Yang, Yuan and Gabriel 1996)(Chakrabarty, Duan and Gabriel 1997)  (Delannoy *et al.* 2005) |

**References**

Astua-Monge G, Minsavage G V., Stall RE *et al.* *Xv4-vrxv4* : A New Gene-for-Gene Interaction Identified Between *Xanthomonas campestris* pv. *Vesicatoria* Race T3 and the Wild Tomato Relative *Lycopersicon pennellii*. *Mol Plant-Microbe Interact* 2000;**13**:1346–55.

Chakrabarty PK, Duan YP, Gabriel DW. Cloning and Characterization of a Member of the *Xanthomonas avr/pth* Gene Family That Evades All Commercially Utilized Cotton *R* Genes in the United States. *Phytopathology* 1997;**87**:1160–7.

Cheong MS, Kirik A, Kim JG *et al.* AvrBsT Acetylates Arabidopsis ACIP1, a Protein that Associates with Microtubules and Is Required for Immunity. *PLoS Pathog* 2014, DOI: 10.1371/journal.ppat.1003952.

Delannoy E, Lyon BR, Marmey P *et al.* Resistance of cotton towards Xanthomonas campestris pv. malvacearum. *Annu Rev Phytopathol* 2005;**43**:63–82.

De Feyter R, Gabriel DW. Use of cloned DNA methylase genes to increase the frequency of transfer of foreign genes into Xanthomonas campestris pv. malvacearum. *J Bacteriol* 1991, DOI: 10.1128/jb.173.20.6421-6427.1991.

Gochez AM, Minsavage G V., Potnis N *et al.* A functional XopAG homologue in Xanthomonas fuscans pv. aurantifolii strain C limits host range. *Plant Pathol* 2015, DOI: 10.1111/ppa.12361.

Gu K, Yang B, Tian D *et al.* R gene expression induced by a type-III effector triggers disease resistance in rice. *Nature* 2005, DOI: 10.1038/nature03630.

Gupta MK, Nathawat R, Sinha D *et al.* Mutations in the Predicted Active Site of Xanthomonas oryzae pv. oryzae XopQ Differentially Affect Virulence, Suppression of Host Innate Immunity, and Induction of the HR in a Nonhost Plant . *Mol Plant-Microbe Interact* 2014, DOI: 10.1094/mpmi-09-14-0288-r.

Horvath DM, Stall RE, Jones JB *et al.* Transgenic resistance confers effective field level control of bacterial spot disease in tomato. *PLoS One* 2012, DOI: 10.1371/journal.pone.0042036.

Hu K, Cao J, Zhang J *et al.* Improvement of multiple agronomic traits by a disease resistance gene via cell wall reinforcement. *Nat Plants* 2017, DOI: 10.1038/nplants.2017.9.

Ji Z, Ji C, Liu B *et al.* Interfering TAL effectors of Xanthomonas oryzae neutralize R-gene-mediated plant disease resistance. *Nat Commun* 2016, DOI: 10.1038/ncomms13435.

Kearney B, Staskawicz BJ. Widespread distribution and fitness contribution of Xanthomonas campestris avirulence gene avrBs2. *Nature* 1990;**346**:385–6.

Kim NH, Choi HW, Hwang BK. *Xanthomonas campestris* pv. *vesicatoria* Effector AvrBsT Induces Cell Death in Pepper, but Suppresses Defense Responses in Tomato. *Mol Plant-Microbe Interact* 2010;**23**:1069–82.

Mendes BMJ, Cardoso SC, Boscariol-Camargo RL *et al.* Reduction in susceptibility to Xanthomonas axonopodis pv. citri in transgenic Citrus sinensis expressing the rice Xa21 gene. *Plant Pathol* 2010, DOI: 10.1111/j.1365-3059.2009.02148.x.

Potnis N, Minsavage G, Smith JK *et al.* Avirulence Proteins AvrBs7 from Xanthomonas gardneri and AvrBs1.1 from Xanthomonas euvesicatoria Contribute to a Novel Gene-for-Gene Interaction in Pepper . *Mol Plant-Microbe Interact* 2011, DOI: 10.1094/mpmi-08-11-0205.

Pruitt RN, Schwessinger B, Joe A *et al.* The rice immune receptor XA21 recognizes a tyrosine-sulfated protein from a Gram-negative bacterium. *Sci Adv* 2015, DOI: 10.1126/sciadv.1500245.

Römer P, Hahn S, Jordan T *et al.* Plant pathogen recognition mediated by promoter activation of the pepper Bs3 resistance gene. *Science (80- )* 2007, DOI: 10.1126/science.1144958.

Ronald PC, Staskawicz BJ. The avirulence gene avrBs1 from Xanthomonas campestris pv. vesicatoria encodes a 50-kD protein. *Mol Plant Microbe Interact* 1988;**1**:191–8.

Schornack S, Ballvora A, Gürlebeck D *et al.* The tomato resistance protein Bs4 is a predicted non-nuclear TIR-NB-LRR protein that mediates defense responses to severely truncated derivatives of AvrBs4 and overexpressed AvrBs3. *Plant J* 2004, DOI: 10.1046/j.1365-313X.2003.01937.x.

Schornack S, Minsavage G V., Stall RE *et al.* Characterization of AvrHah1, a novel AvrBs3-like effector from Xanthomonas gardneri with virulence and avirulence activity. *New Phytol* 2008, DOI: 10.1111/j.1469-8137.2008.02487.x.

Schornack S, Peter K, Bonas U *et al.* Expression Levels of *avrBs3* -Like Genes Affect Recognition Specificity in Tomato *Bs4* - But Not in Pepper *Bs3* -Mediated Perception. *Mol Plant-Microbe Interact* 2005;**18**:1215–25.

Schwartz AR, Potnis N, Timilsina S *et al.* Phylogenomics of Xanthomonas field strains infecting pepper and tomato reveals diversity in effector repertoires and identifies determinants of host specificity. *Front Microbiol* 2015, DOI: 10.3389/fmicb.2015.00535.

Sendín LN, Orce IG, Gómez RL *et al.* Inducible expression of Bs2 R gene from Capsicum chacoense in sweet orange (Citrus sinensis L. Osbeck) confers enhanced resistance to citrus canker disease. *Plant Mol Biol* 2017;**93**:607–21.

Song WY, Wang GL, Chen LL *et al.* A receptor kinase-like protein encoded by the rice disease resistance gene, Xa21. *Science (80- )* 1995, DOI: 10.1126/science.270.5243.1804.

Strauss T, van Poecke RMP, Strauss A *et al.* RNA-seq pinpoints a Xanthomonas TAL-effector activated resistance gene in a large-crop genome. *Proc Natl Acad Sci* 2012, DOI: 10.1073/pnas.1212415109.

Sun X, Cao Y, Yang Z *et al.* Xa26, a gene conferring resistance to Xanthomonas oryzae pv. oryzae in rice, encodes an LRR receptor kinase-like protein. *Plant J* 2004, DOI: 10.1046/j.1365-313X.2003.01976.x.

Tai TH, Dahlbeck D, Clark ET *et al.* Expression of the Bs2 pepper gene confers resistance to bacterial spot disease in tomato. *Proc Natl Acad Sci U S A* 1999;**96**:14153–8.

Tian D, Wang J, Zeng X *et al.* The Rice TAL Effector-Dependent Resistance Protein XA10 Triggers Cell Death and Calcium Depletion in the Endoplasmic Reticulum. *Plant Cell* 2014, DOI: 10.1105/tpc.113.119255.

Tripathi JN, Lorenzen J, Bahar O *et al.* Transgenic expression of the rice Xa21 pattern-recognition receptor in banana (Musa sp.) confers resistance to Xanthomonas campestris pv. musacearum. *Plant Biotechnol J* 2014;**12**:663–73.

Van Den Ackerveken G, Marois E, Bonas U. Recognition of the bacterial avirulence protein AvrBs3 occurs inside the host plant cell. *Cell* 1996, DOI: 10.1016/S0092-8674(00)81825-5.

Wang C, Zhang X, Fan Y *et al.* XA23 Is an executor r protein and confers broad-spectrum disease resistance in rice. *Mol Plant* 2015, DOI: 10.1016/j.molp.2014.10.010.

Wang H, Hutton SF, Robbins MD *et al.* Molecular Mapping of Hypersensitive Resistance from Tomato ‘Hawaii 7981’ to Xanthomonas perforans Race T3 . *Phytopathology* 2011, DOI: 10.1094/phyto-12-10-0345.

Wang J, Zeng X, Tian D *et al.* The pepper Bs4C proteins are localized to the endoplasmic reticulum (ER) membrane and confer disease resistance to bacterial blight in transgenic rice. *Mol Plant Pathol* 2018, DOI: 10.1111/mpp.12684.

Wang L, Tang X, He C. The bifunctional effector AvrXccC of Xanthomonas campestris pv. campestris requires plasma membrane-anchoring for host recognition. *Mol Plant Pathol* 2007, DOI: 10.1111/j.1364-3703.2007.00409.x.

Whalen MC, Wang JF, Carland FM *et al.* Avirulence gene avrRxv from Xanthomonas campestris pv. vesicatoria specifies resistance on tomato line Hawaii 7998. *Mol Plant Microbe Interact* 1993;**6**:616–27.

Xu RQ, Blanvillain S, Feng JX *et al.* AvrACXcc8004, a type III effector with a leucine-rich repeat domain from Xanthomonas campestris pathovar campestris confers avirulence in vascular tissues of Arabidopsis thaliana ecotype Col-0. *J Bacteriol* 2008, DOI: 10.1128/JB.00978-07.

Yang Y, Yuan Q, Gabriel DW. Watersoaking Functlon(s) of XcmH1005 Are Redundantly Encoded by Members of the *Xanthomonas avr/pth* Gene Family. *Mol Plant-Microbe Interact* 1996;**9**:105.

Yoshimura S, Yamanouchi U, Katayose Y *et al.* Expression of Xa1, a bacterial blight-resistance gene in rice, is induced by bacterial inoculation. *Proc Natl Acad Sci U S A* 1998;**95**:1663–8.

Zhao B, Ardales EY, Raymundo A *et al.* The *avrRxo1* Gene from the Rice Pathogen *Xanthomonas oryzae* pv. *oryzicola* Confers a Nonhost Defense Reaction on Maize with Resistance Gene *Rxo1*. *Mol Plant-Microbe Interact* 2004a;**17**:771–9.

Zhao BY, Ardales E, Brasset E *et al.* The Rxo1/Rba1 locus of maize controls resistance reactions to pathogenic and non-host bacteria. *Theor Appl Genet* 2004b, DOI: 10.1007/s00122-004-1623-y.
